# A *Bacillus*-based direct-fed microbial mixture remodels the gut microbiome to augment the respiratory health of *Salmonella*-infected pigs

**DOI:** 10.1128/aem.00972-26

**Published:** 2026-07-10

**Authors:** Taylor Putman, Ahmed M. Abdel-Hamid, Elizabeth Galbraith, Patrick Schimmel, Hyewon Kim, Taro Yasuma, Manal A. B. Alhawsawi, Kingsley A. Boateng, Jessica Holmes, Megan Duersteler, Corina N. D'Alessandro-Gabazza, Hajime Fujimoto, Tetsu Kobayashi, Kimberly K. O. Walden, Gloria Rendon, Christopher J. Fields, Federico A. Zuckermann, Roderick I. Mackie, Sona Son, Kyle R. Leistikow, Esteban C. Gabazza, Michael R. King, Isaac Cann

**Affiliations:** 1 Department of Animal Science, University of Illinois Urbana-Champaign 166978, Urbana, Illinois, USA; 2 Carl R. Woese Institute for Genomic Biology (Microbiome Metabolic Engineering), University of Illinois Urbana-Champaign 124518, Urbana, Illinois, USA; 3 Department of Botany and Microbiology, Faculty of Science, Minia University 110162, El-Minia, Egypt; 4 Microbial Discovery Group 710904 , Oak Creek, Wisconsin, USA; 5 Department of Immunology, Mie University Faculty and Graduate School of Medicine 12946, Tsu, Mie, Japan; 6 Department of Diabetes and Endocrinology, Mie University Faculty and Graduate School of Medicine 12946, Tsu, Mie, Japan; 7 Division of Nutritional Sciences, University of Illinois Urbana-Champaign 242192, Urbana, Illinois, USA; 8 Clinical Nutrition Department, College of Applied Medical Sciences, University of Hafr Al Batin 48098, Hafar Al Batin, Saudi Arabia; 9 Roy J. Carver Biotechnology Center, The University of Illinois at Urbana-Champaign 14589, Urbana, Illinois, USA; 10 Microbiome Research Center, Mie University 12946, Tsu, Mie, Japan; 11 Department of Pulmonary and Critical Care Medicine, Mie University Faculty and Graduate School of Medicine 12946, Tsu, Mie, Japan; 12 Department of Pathobiology, College of Veterinary Medicine, University of Illinois Urbana-Champaign 70154, Urbana, Illinois, USA; 13 Center for East Asian and Pacific Studies, University of Illinois Urbana-Champaign 14589, Urbana, Illinois, USA; 14 Department of Microbiology, University of Illinois Urbana-Champaign 189313, Urbana, Illinois, USA; Michigan State University, East Lansing, Michigan, USA

**Keywords:** direct-fed microbials, pig lung health

## Abstract

Commercial pork production is practiced worldwide and represents a major source of protein for global populations. Pigs, however, are plagued by various diseases that affect their productivity. A common practice is to administer antibiotics in the feed to reduce infections and promote growth. However, antibiotic utilization in pig production has been identified as a source of spread of antibiotic resistance genes, prompting the need for antibiotic alternatives in swine production. *Salmonella enterica* serotype Choleraesuis and porcine reproductive and respiratory syndrome virus (PRRSV) are two disease agents with a significant impact on the pork industry. In this study, we designed experiments to test the hypothesis that a *Bacillus*-based direct-fed microbial (DFM) cocktail will alleviate the impact of *Salmonella* infection alone or in combination with PRRSV. Both single and dual infections resulted in shifts in the cecal microbiota from that of the Control group, with administration of the DFM dampening this effect, especially in the *Salmonella*-infected group. In the absence of the DFM, the infected pigs exhibited gross changes in the lungs, including tissue hepatization. Significantly, the DFM application suppressed the lesions in the lungs of *Salmonella*-only infected pigs. Using metagenome-assembled genomes, we found that DFM administration to *Salmonella*-only infected pigs led to cecal microbiota enriched in the potential to produce immune-stimulating short-chain fatty acids and naturally occurring antimicrobials, including peptides. The putative antimicrobial peptides derived from this study, upon biochemical characterization, could lead to their application as novel antimicrobials in animal agriculture and health.

**IMPORTANCE:**

Antibiotics, as feed additives, have been integral to commercial pork production. Their use, however, has fostered the spread of antibiotic resistance genes in the environment. In this study, we explored the use of a mixture of naturally occurring bacteria, comprising species of the genus *Bacillus*, as an alternative to antibiotics in the pig diet. The bacterial mixture reversed disease lesions in the lungs of pigs infected with *Salmonella enterica* serotype Choleraesuis, a bacterium that causes severe disease in commercial pigs. Our findings suggest that applying the bacterial mixture to the *Salmonella*-infected pigs shifts the microbes in the gut to a community that is endowed with antimicrobials that mitigate the effects of *Salmonella* infection. We present data showing the novelty of putative antimicrobials discovered in the present study and postulate that their characterization will yield new antimicrobials that can be used in different sectors of animal production and health. PRRSV was included in the study to model a common bacterial-viral co-infection in swine, as it exacerbates disease severity. This design allowed assessment of whether *Bacillus*-based DFM could improve outcomes along the gut–lung axis under realistic co-infection conditions.

## INTRODUCTION

The growth in demand for animal-origin foods to meet the dietary needs of a growing world population has led to intensive farming to increase meat production and availability (1). However, a significant constraint to intensive livestock production is the incidence and spread of disease due to close animal proximity (2). Most livestock diseases affect animal health and decrease productivity, with some of these diseases, collectively known as zoonotic diseases, also being transmissible to humans (2). It is, therefore, common practice for farmers to use antibiotics to inhibit the pathogens that cause these diseases and limit the spread of animal infections. Long-term overuse and misuse of antibiotics in animal agriculture and veterinary and human medicine have contributed to the rise of multidrug-resistant pathogens (3, 4), the spread of which reduces the efficacy of antibiotic treatment (5). Alternative strategies to antibiotic use are, therefore, urgently needed to reduce the prevalence and global spread of antimicrobial resistance (AMR).

Direct-fed microbials (DFMs) are dietary supplements containing viable naturally occurring microorganisms that, when fed to the host animal at precise concentrations, generate health benefits. The use of DFMs in livestock production provides an ideal alternative to antibiotic usage to reduce their negative downstream impacts (6). Thus, research focused on investigating DFMs or probiotics as antibiotic alternatives has grown exponentially since the early 2000s (6), with some preparations shown to prevent and treat a variety of diseases directly through competitive interactions with pathogens (7).

Diverse bacteria have been harnessed in DFM applications, and members of the genera *Lactobacillus*, *Bifidobacterium*, *Enterococcus*, *Streptococcus*, *Pediococcus*, and *Bacillus* are among the most commonly used in animal production (8). However, among this group, the genus *Bacillus* has generated heightened interest in the livestock industry due to its capacity to sporulate and produce a variety of antimicrobials and immune-modulating signaling peptides (6). A recent study examined the impact of providing weanling pigs a *Bacillus*-based DFM on the disease syndrome resulting from infection with either *Salmonella enterica* serotype Choleraesuis alone or in combination with porcine reproductive and respiratory syndrome virus (PRRSV) (9). These results showed that the DFM reduced the pathogenic synergy of coinfection by the bacterium and the virus, increased levels of pulmonary cytokines associated with viral clearance, and increased systemic gene expression of innate immune markers, suggesting a heightened readiness of the innate immune system to modulate infections in the respiratory tract (9). These findings raised new questions about whether the immunomodulatory effects of this *Bacillus*-based DFM may be associated with changes in the gastrointestinal or pulmonary microbial communities of the pig.

Advancements in high-throughput next-generation sequencing (NGS) technologies have improved our understanding of microbial community characteristics, including diversity and abundance, and the temporal, spatial, and evolutionary dynamics of the community (10). Applications of 16S rRNA gene sequencing and shotgun metagenomics have been instrumental in profiling microbial diversity, reconstructing metagenome-assembled genomes (MAGs), and identifying putative functional pathways (11, 12). These approaches have strengthened the understanding of intestinal microbiomes and their role in health and disease. Here, we use NGS and other approaches to better understand how a *Bacillus*-based DFM impacts the bacterial communities in the lower gastrointestinal tract and lungs of pigs challenged with either *Salmonella enterica* serotype Choleraesuis alone or in combination with PRRSV. We further examine potential changes in the microbiome that might have reversed the significant changes in lung histology observed in pigs challenged with *S. enterica* serotype Choleraesuis in the present study. Both *Salmonella* and PRRSV infections impose major costs on the pork industry, and DFMs that alleviate their incidence and/or pathogenic traits will yield both economic and public health benefits. We hypothesized that *Bacillus*-based DFM would modulate the gut microbiome to enhance resistance to *Salmonella* infection and reduce disease severity in the single and co-infection models.

## RESULTS

### Both infection and DFM application impact pig gut microbiota diversity matrices

Pigs were assigned to five groups (Control, *Salmonella* only, *Salmonella* + DFM, *Salmonella*/PRRSV, and *Salmonella*/PRRSV + DFM (Table S1). Samples from the cecum, a primary site of microbial fermentation in pigs, and lungs were analyzed by 16S rRNA gene sequencing, metagenomics, and histopathology to gain insight into how the treatments impacted the physiology of the animals. To enhance the reliability of our results, we initially implemented alpha rarefaction analysis, and the plateauing of the rarefaction curves for the bacterial diversity in both the digesta and mucosal samples suggested that all dominant species were successfully captured in each sample via 16S rRNA gene sequencing (Fig. S1). Infected pigs developed expected respiratory symptoms and diarrhea, confirming successful infection. *Salmonella* was recovered from relevant tissues in infected groups but was absent in controls.

Faith phylogenetic diversity (Faith PD), richness, and phylogenetic distance estimates were calculated from filtered and non-normalized feature data using phyloseq and ggplot packages (Fig. S2). For the digesta, the lowest diversity estimate occurred in the double infection with the DFM group (Fig. S2A; *P* < 0.05). Meanwhile, the diversity estimates in the digesta samples from the Control group were much less variable than in the samples from other experimental groups, especially in the infection groups without DFM. In the mucosal samples, we observed the least variability again in the Control group, and this group presented the lowest average estimated richness (Fig. S2A). Furthermore, the *Salmonella* + DFM and the double infection group (*Salmonella* + PRRSV) had a statistically significantly higher estimated richness compared to the Control group (*P* < 0.05) in mucosal samples, whereas in the digesta samples, other than the *Salmonella*/PRRSV + DFM group, the estimated richness for the samples was not statistically different (*P* > 0.05). In Fig. S2B, it was observed that the estimated species richness is higher in the absence of the DFM; however, these differences were not statistically significant (*P* > 0.05). The mucosa samples across all pigs were found to exhibit a statistically significantly higher estimated species richness compared to the digesta samples (Fig. S2C; *P* < 0.001). Although it is common that digesta have higher richness than mucosa, higher richness in mucosa has also been reported (13, 14).

### Gut microbiota composition is affected by infection state and DFM administration

**Fig 1:**
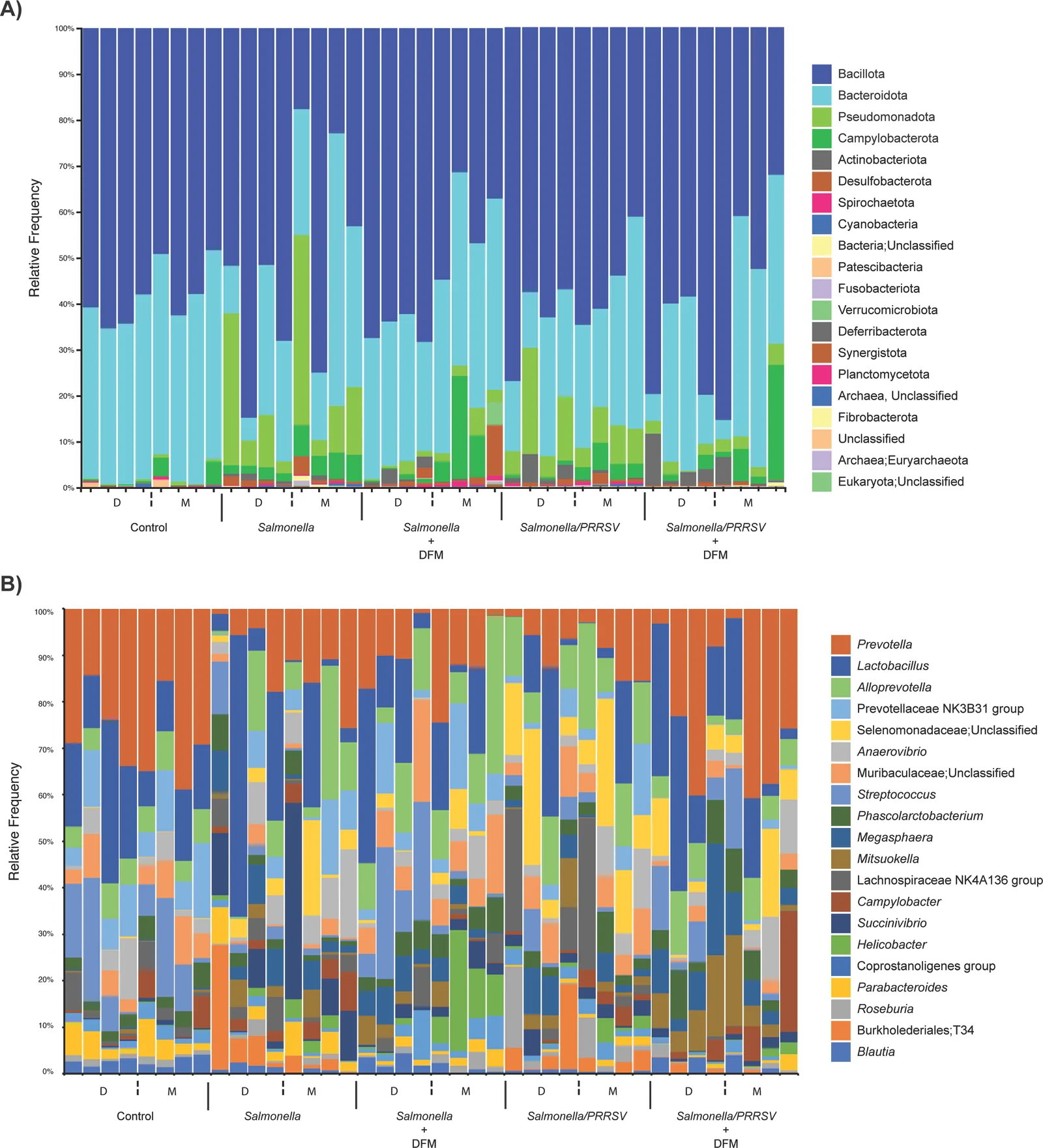
Stacked bar charts showing relative abundance. Relative abundance (%) of (**A**) phyla and (**B**) top 20 genera based on 16S rRNA gene amplicon sequencing from digesta (D) and mucosa (M) samples from the four experimental groups of pigs and a set of control pigs. The bar height for each color represents the relative abundance of a bacterial taxon.

At the phylum level (Fig. 1A), the Control group exhibited a consistent bacterial composition in both the digesta and mucosa of the cecum, with about 60% of the bacterial population belonging to Bacillota (formerly Firmicutes) and about 40% belonging to the Bacteroidota. By contrast, pigs infected with either *Salmonella* or *Salmonella*/PRRSV exhibited higher relative abundance of other bacterial phyla, mainly Pseudomonadota and Campylobacterota. The presence of the DFM, that is, the *Salmonella +* DFM and *Salmonella*/PRRSV + DFM groups, led to a lower relative abundance of the Pseudomonadota (Fig. 1A).

At the genus level (Fig. 1B), the *Salmonella* group exhibited a higher relative abundance of *Succinivibrio* and *Burkholderiales*, and interestingly, the presence of the DFM led to a higher relative abundance of *Helicobacter*-related reads in the mucosa samples. Furthermore, a group of bacteria designated unclassified *Selenomonadaceae* was relatively less abundant in the Control group compared to any infected group of pigs, but especially in the *Salmonella*/PRRSV co-infection group. However, the DFM application tended to decrease the relative abundance of this group of bacteria compared to infected pigs without DFM, especially in the digesta samples. The presence of the DFM also appeared to restore the *Prevotella* in the *Salmonella*/PRRSV + DFM group to the high levels observed in the Control animals. Furthermore, the application of the DFM restored the genus *Streptococcus* to a similar abundance as in the Control group, especially in the digesta of some of the pigs in the *Salmonella +* DFM group. Importantly, 16S rRNA gene sequencing revealed the presence of *Salmonella enterica* in the treatment groups, whereas it was undetectable in the Control group (Fig. S3).

Principal Coordinates Analysis (PCoA) was performed on a Bray–Curtis dissimilarity matrix for samples separated by all four experimental and Control groups (Fig. S4A). Further analyses were then based on the Control group and the presence or absence of DFM application (Fig. S4B). The first two principal components constituted a total of 32.7% of the total variation. Control group samples from both the digesta and mucosa clustered together, indicating their similarity, corroborating our earlier findings in Fig. S2A. The *Salmonella*-challenged group digesta samples showed a shift away from the Control group without a clear clustering. The *Salmonella* + DFM group generally clustered together, with one outlier, and shifted closer to the Control group (compared to the *Salmonella* group). Similarly, the *Salmonella* + PRRSV group did not show clear clustering; however, in the presence of the DFM, clustering was observed at the top-right quadrant. In the mucosa samples, the *Salmonella*-challenged group generally clustered in the bottom-right quadrant, and while the presence of the DFM led to shifts toward the Control group, a clear clustering was not observed. Clustering was, however, observed for the mucosa samples from the *Salmonella* + PRRSV group, and except for one outlier, DFM application led to a shift and, importantly, clustering in the upper-right quadrant, as the samples from the digesta are of the same group. Although there was no clear clustering of all samples, application of the DFM cocktail tended to shift both the mucosa and digesta samples toward the Control group (Fig. S4B).

### Application of the DFM has a significant effect on the lung pathology of the pigs in the *Salmonella* group

**Fig 2:**
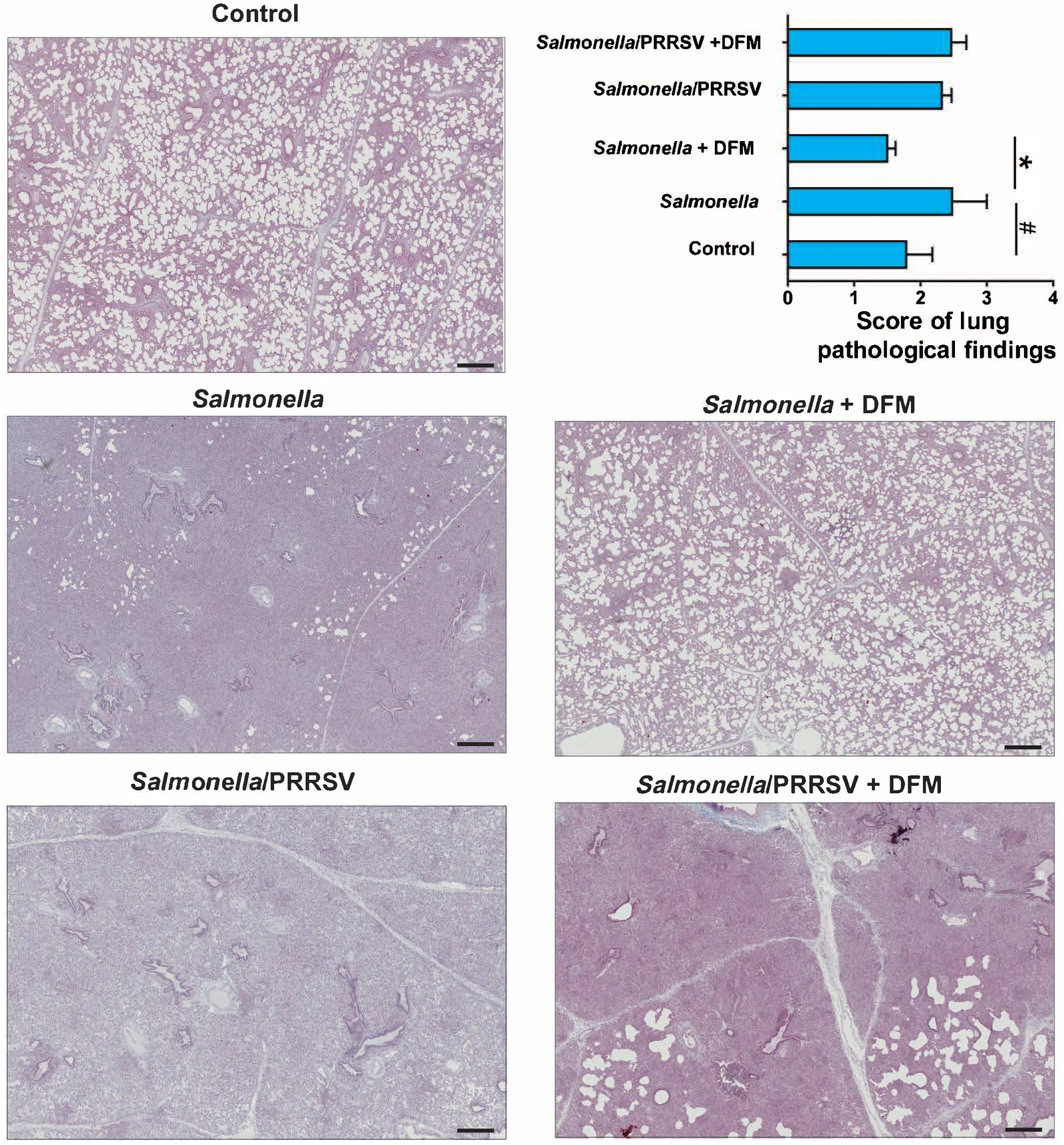
Lung pathological findings in pigs. Pigs were allocated to five experimental groups: a control group (Control), a group with *Salmonella enterica* infection (*Salmonella*), a group with *Salmonella enterica* infection and DFM treatment (*Salmonella* + DFM), a group with both *Salmonella enterica* and PRRSV infection (*Salmonella/*PRRSV), and a group with both *Salmonella enterica* and PRRSV infection and DFM treatment (Salmonella/PRRSV + DFM). Lung sections were stained with Masson’s trichrome, and seven experts in the field of pulmonary disease, blinded to the treatments, scored the lung pathological findings based on the following criteria: Score 0: alveolar wall thickening or lung consolidation in 0%–25% of the total lung field with the absence of intra-alveolar debris, fluid, or interlobular septal thickening; Score 1: alveolar wall thickening or lung consolidation in 25%–50% of the total lung field with positive or negative findings of intra-alveolar debris, fluid, or interlobular septal thickening; Score 2: alveolar wall thickening or lung consolidation in 50%–75% of the total lung field with mild grade (+) of intra-alveolar debris, fluid, or interlobular septal thickening; Score 3: alveolar wall thickening or lung consolidation in 75%–100% of the total lung field with severe grade (++) of intra-alveolar debris, fluid, or interlobular septal thickening. Data are expressed as the mean S.E.M. Scale bars indicate 500 μm. Statistical analysis was performed using analysis of variance with Fisher’s least significant difference. #*P* = 0.1, *P < 0.05.

Based on the observation that the DFM application shifted the microbial communities in the samples toward those of the Control group, we sought to determine how the changes affected by the DFM impacted the physiology of the animals. We focused on the lung physiology, as both *Salmonella enterica* serotype Choleraesuis and PRRSV are known to target the lungs and cause significant respiratory pathology in swine. In Fig. 2, we present the pathology of the lungs and scoring of stained lung tissues of the pigs from the Control and the various experimental groups. Experts in lung pathology, blinded to the groups, scored the *Salmonella* + DFM group lung histology significantly differently from the *Salmonella* group (*P* < 0.05, bar graph). In the *Salmonella*-challenged group, the foregoing lung symptoms were, on average, assessed at about 75% of the lung field, and importantly, a severe grade of lung tissue damage was observed. In contrast, in the Control and *Salmonella* + DFM groups, approximately 50% of the total lung field was considered to have alveolar wall thickening or lung consolidation, with only a mild or lower grade of intra-alveolar debris, fluid, or interlobular septal thickening. Across all the results, infection with *Salmonella* or the combination of *Salmonella* and PRRSV led to hepatization of the tissue (Fig. 2, see *Salmonella* and *Salmonella*/PRRSV). Application of the DFM in the double-infection group led to changes in staining color and changes in tissue structure, even though these were not reflected in the score by the experts. Importantly, hepatization in the *Salmonella* treatment group was completely prevented by the application of the DFM (Fig. 2; see *Salmonella* + DFM).

The lung harbors its own microbiome (15). Therefore, we used Shoreline amplicon sequencing (Shoreline Biome, United States) to determine whether differences existed in the lung microbiota of the different groups. This method is suitable for PCR amplification from low amounts of DNA and yields full-length 16S rRNA gene sequences, helping to identify organisms at the species level. The amplicon sequences suggested a bacterial community dominated by a large group with an unassigned genus, strains of *Escherichia coli*, *Pseudomonas yamanorum*, *Burkholderia contaminans*, *Staphylococcus haemolyticus, Megasphaera elsdenii,* and, in a few cases, *Streptococcus suis* and *Bacillus pseudofirmus* (Fig. S5A). Using a heatmap approach, we searched for potential differences or signatures that may explain the dramatic changes in the lung histology; however, the abundances of the different bacteria did not follow any clear pattern (Fig. S5B).

### Discriminants of gut microbiota reveal taxa associated with infection and DFM

**Fig 3:**
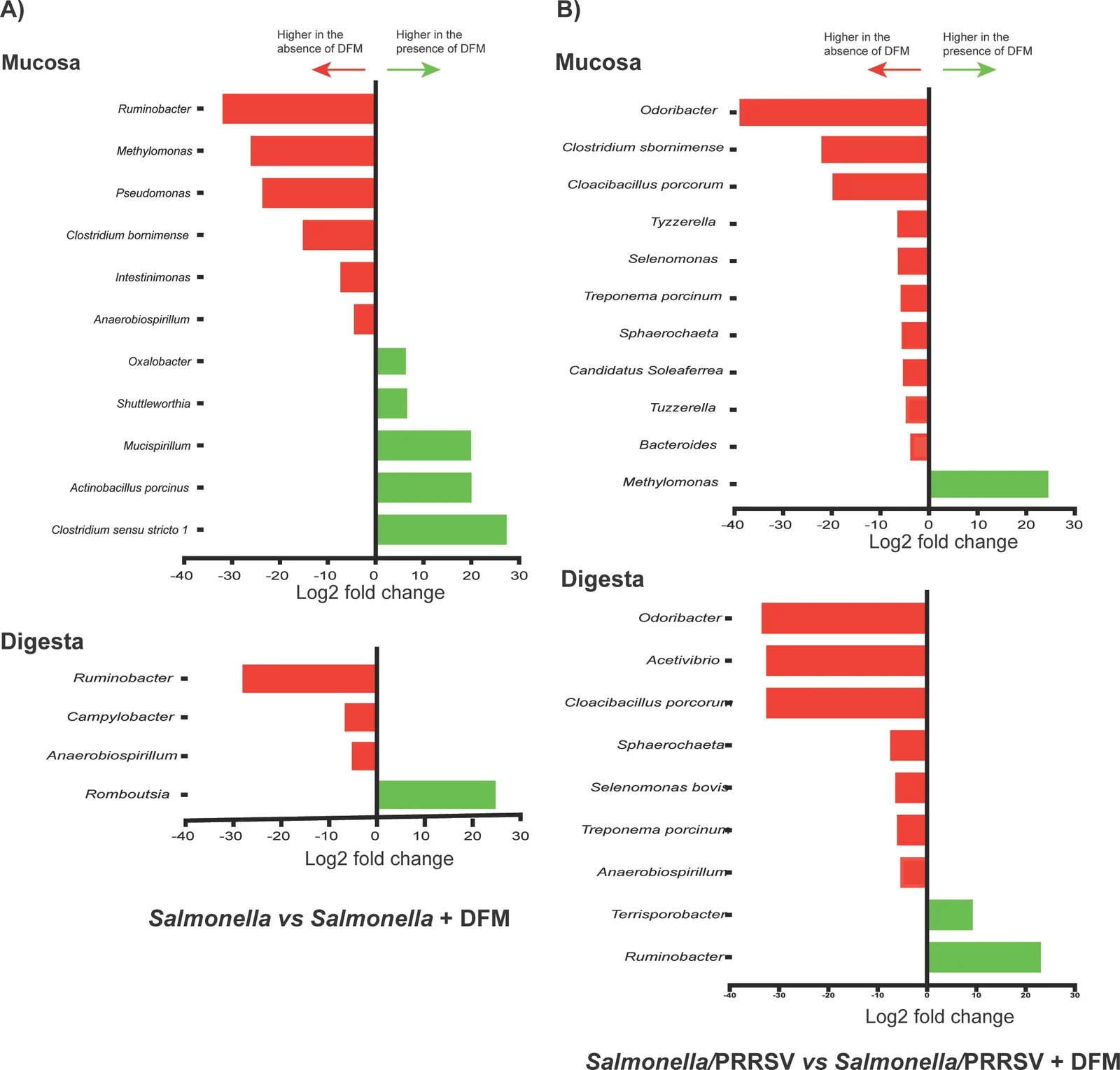
Differential abundance in the presence and absence of DFM. Log2 fold change for the abundance of bacteria (at least the genus is identified) when comparing the microbial population in (**A**) pigs challenged with *Salmonella* and treated with the DFM vs. pigs challenged with *Salmonella* in the absence of DFM and (**B**) pigs challenged with both *Salmonella* and PRRSV and treated with the DFM vs. pigs challenged with *Salmonella* plus PRRSV in the absence of DFM. Positive log2 fold change (green) indicates bacteria that are higher in the presence of DFM, whereas negative log2 fold change (red) indicates bacteria that are higher in the absence of DFM. Only significant data with adjusted *P* < 0.05 are presented.

Our analysis of the differences in the microbiota resident in the lung did not yield results that might explain the observed changes in lung histology. Therefore, differential abundances were calculated for the cecal digesta and mucosal samples to identify the taxa that differed significantly between animals administered with or without the DFM (Fig. 3). Pigs infected with *Salmonella* exhibited higher levels of *Ruminobacter* and *Anaerobiospirillum* in both mucosa and digesta. In addition, *Methylomonas*, *Pseudomonas*, *Clostridium bomimense*, and *Intestinimonas* were higher in the mucosa, and *Campylobacter* was more abundant in the digesta (Fig. 3A). Importantly, DFM application to the *Salmonella*-challenged animals led to higher abundances of *Clostridium sensu stricto* 1, *Actinobacillus*, *Mucispirillum*, *Shuttleworthia,* and *Oxalobacter* in mucosa and *Romboutsia* in digesta (Fig. 3A; *P* < 0.05). Notably, for the double infection group in the absence of the DFM, several bacteria, including members of the genera *Odoribacter* and *Sphaerochaeta* and strains of *Cloacibacillus porcorum, Selenomonas bovis,* and *Treponema porcinum,* were found to be enriched in both mucosa and digesta. In addition, *Clostridium bornimense*, *Tyzzerella*, Candidatus Soleaferrea, *Tuzzerella,* and *Bacteroides* were higher in the mucosa, whereas *Acetivibrio* and *Anaerobiospirillum* were more abundant in the digesta. Application of DFM to the double-infected pigs then led to a higher abundance of members of *Methylomonas* in the mucosa and *Terrisporobacter* and *Ruminobacter* in the digesta (Fig. 3B; *P* < 0.05). In Fig. S6, we present bacterial groups that did not have genus classification but were nevertheless significantly associated with infection state or DFM supplementation, highlighting that poorly characterized lineages of the pig gut microbiome also respond to DFM treatment and may contribute to functional differences.

### Assembly of metagenomes to gain insights into the genetic potential of the microbial communities

The most significant impact of the DFM application, based on our lung histology analysis, was its effect on the *Salmonella*-infected pigs (Fig. 2). Therefore, we carried out further analyses, using metagenomics, to probe any shifts that might have occurred in the genetic makeup of the cecal microbial communities (microbiomes) due to either the *Salmonella* or the *Salmonella* + DFM administration. Unexpectedly, a significant number of reads from three pigs aligned with the pig reference genome. The sample IDs were 172, 173, and 181. Two samples (IDs 172 and 173) were derived from the *Salmonella* group, and one sample (ID 181) was from the *Salmonella* + DFM group (Tables S2 and S3). Furthermore, one of the pigs from the *Salmonella* group (sample 172) appeared to be colonized by a parasite (*Leishmania mexicana,* an eukaryote). Reads from this parasite also resulted in a significant amount of sequence information being removed from this sample before our final analysis. As expected, the Illumina metagenome assemblies were relatively fragmented. The highest contig length from Illumina assemblies reached only ~0.71 Mbp (N50s ranging from 2.9 to 4.5 kbp), whereas ONP assemblies commonly produced contigs >2 Mbp, with N50 values exceeding 60 kbp. Importantly, the samples that lost a significant amount of reads due to high levels of contaminating host genomic DNA did not yield large contigs and were classified as low-tier data in our comparative analysis of the samples.

### Genomic analyses of MAGs: short-chain fatty acid production, antibiotic resistance, and biosynthetic gene clusters across treatment groups

The quality of the acquired MAGs was assessed by plotting completeness of the MAGs against the contamination levels derived from CheckM (Fig. S7). Quality filtering of the MAGs led to a remainder of 111 bacterial MAGs, with 10 MAGs from the *Salmonella* group (Animal/sample id 3 = 6 MAGs and id 7 = 4 MAGs), 36 from the Control group (Animal/sample id 161 = 7 MAGs, id 162 = 13 MAGs, id 163 = 11 MAGs, and id 164 = 5 MAGs), and 65 from the *Salmonella* + DFM group (Animal/sample id 177 = 21 MAGs, id 179 = 23 MAGs, and id 182 = 21 MAGs). Notably, no MAG was assembled or analyzed from animal/sample ids 172 and 173, derived from the *Salmonella* group, and animal id 181 from the *Salmonella* + DFM group (Tables S2 and S3).

The MAGs were constructed per sample and thus allow the identification of MAGs with identical marker gene sequences across different samples (Data Set S1). Based on the classification of the MAGs, the most common derived from the phylum Bacillota with 78 MAGs, followed by the Bacteroidota with 24 MAGs, a reflection of the dominance of the Bacillota in the gut of the pigs under study (see Fig. 1A). Among the Bacillota, the genus with the highest number of MAGs was *Streptococcus*, and this was followed by *Ruminococcus* and *Eubacterium*, all of which were found in the Control and *Salmonella* + DFM groups. In the Bacteroidota, the most common genus was *Prevotella,* and most of these MAGs were from the *Salmonella* group (Data Set S1).

**Fig 4:**
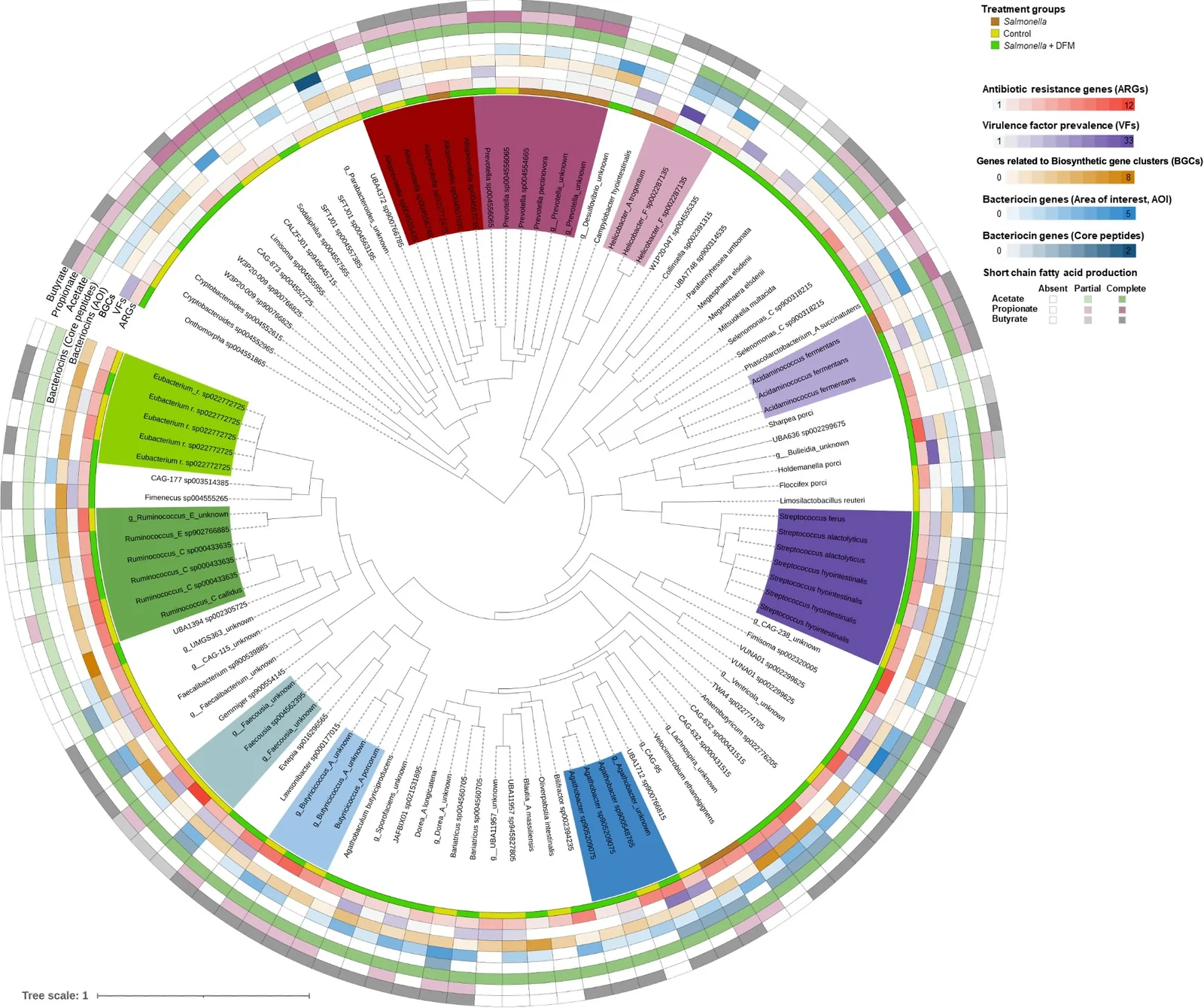
Phylogenetic tree depicting some of the contents of the high-quality MAGs. Occurrence of ARGs, VFs, bacteriocins, presence of pathways related to butyrate, acetate, and propionate production, and finally loci of BGCs are shown on the six outer rings. The inner ring shows the treatment group origin of the genome. Species assignment is from the GTDBTk taxonomic assignment.

For ease of visualization, the phylogenetic tree presenting the results (genetic make-up) from the analyses of the MAGs was annotated with circular heat maps of genes predicted to function in antibiotic resistance (antibiotic resistance genes or ARGs), virulence factor (VF) prevalence, biosynthetic gene clusters (BGCs), bacteriocins (areas of interest [AOI]), bacteriocin core peptides, and the short-chain fatty acids (SCFAs), acetate, propionate, and butyrate (Fig. 4). The metadata for the phylogenetic tree annotation are found in Data Set S1. The ARGs were most common in the Control group, with an average of 4.9 ARGs per MAG. This was higher than the average of 4.3 found in the *Salmonella* + DFM group, which was higher than the *Salmonella* group, where the average was 3.4. Significantly, we found, in some MAGs, as many as 10 ARGs, and these were either from the Control (*Faecousia* sp004562395 MAG) or the *Salmonella* + DFM group (*Butyricoccus A, Bulleidia, Ventricola*, and CAG-238 MAGs). In the analysis of the VFs, a MAG was annotated as *Helicobacter* sp. (_F sp002287135 or MAG 179 _maxbin_.040.fa) and assembled from a *Salmonella* + DFM group pig (id 179), which contained the highest number (*n* = 33). The relative occurrence of VF in the MAGs was assessed as a level of pathogenic capacity between the treatment arms. The applied database (http://www.mgc.ac.cn/VFs/main.htm) included genes involved in adherence, effector delivery systems, motility, exotoxins, exoenzymes, immune modulation, and biofilm formation. Overall, the VFs were the highest in MAGs from the *Salmonella* + DFM group and occurred at an average of 5.9 per MAG, while in the Control and the *Salmonella* groups, they were found at 1.7 and 2.8, respectively, per MAG (Data Set S1).

The MAGs were further analyzed for the presence, partial presence, or absence of pathways involved in the production of the SCFAs acetate, propionate, and butyrate. Our analyses showed a trend where the *Salmonella* + DFM led to an increased number of MAGs harboring complete SCFA pathways, particularly for butyrate and acetate (Data Set S2). Thus, in the butyrate pathway, the *Salmonella* + DFM group had 36 MAGs with full pathways, compared with 11 in the Control and 8 in the *Salmonella* groups. Similarly, in the acetate pathways, from the *Salmonella* + DFM group, 50 MAGs contained full pathways, compared with 27 in the Control and 9 in the *Salmonella* groups. While the *Salmonella* + DFM group yielded MAGs and potentially microbes with complete pathways for butyrate and acetate, a greater number of MAGs presented only partial propionate pathways across the different groups. In the *Salmonella* + DFM group, we identified 18 MAGs with partial propionate pathways, compared with 10 in the Control and 3 in the *Salmonella* group, respectively. Similarly, a higher number of MAGs with full propionate pathways were detected in the *Salmonella* + DFM group (seven total) and the Control group (six total) than in the *Salmonella* group (two total).

We used the online BGC detection tool antiSMASH to analyze the MAGs, with the resulting identification of a total of 251 BGCs in the 111 MAGs obtained from the pig samples (Data Set S3). Among these MAGs, the most prevalent BGCs were associated with ranthipeptide biosynthesis (*n* = 69), a new class of antibiotics. After the ranthipeptides, the next most prevalent BGCs were another antibiotic group, the ribosomally synthesized and post-translationally modified peptide (RiPP) precursor peptide-recognition element (RRE)-containing clusters (*n* = 55), which were then followed by cyclic-lactone-autoinducer biosynthesis clusters (*n* = 51), which function in quorum-sensing.

In the Control group, we observed that BGCs related to cyclic-lactone-autoinducer biosynthesis (*n*=19) were exclusively present in the MAGs predicted to come from the Bacillota and absent in those of the Bacteroidota. From the Control group, *Gemmiger* sp900554145 and *Ruminococcus*_C sp000433635 contained the most cyclic-lactone-autoinducer biosynthesis-related BGCs of 4 and 3, respectively. In the *Salmonella* group, only one BGC related to cyclic-lactone-autoinducer biosynthesis was identified, and it was in a MAG designated Lachnospiraceae (CAG-95). The MAGs from the *Salmonella* + DFM group showed a large number of BGCs associated with cyclic-lactone-autoinducer biosynthesis (*n*=31), with each MAG derived from the Bacillota. Within these MAGs, UBA1394 sp002305725 exhibited the largest number of 6, followed by a *Lachnospira* sp. MAG with 5, *Fimenecus* sp004555265 with 3, and *Ruminococcus*_C sp000433635 also with 3. The *Ruminococcus*_C sp000433635, with three cyclic-lactone-autoinducer BGCs found in the Control group, appeared to be present also in the *Salmonella* + DFM group.

### Putative bacteriocin gene clusters are highly enriched in the *Salmonella* + DFM group

We next used BAGEL4, a web server developed for mining bacterial metagenomic data for bacteriocins and RiPPS (16). This tool allowed us to delve deeper into the MAGs to determine whether we can gain a clearer insight into changes in the gut microbiome that might have resulted in the differences in the lung histology of the *Salmonella* and the *Salmonella* + DFM groups. Using this approach, 134 putative bacteriocin gene clusters (pbGCs) were delineated in the 111 assembled MAGs. The pbGCs were defined as gene clusters encoding a Bacteriocin-like peptide (Blp)U, bottromycin, bovicin_255_peptide, enterolysin _A, lanthipeptide class II, Linear Azole-containing Peptides (LAPs), lasso_peptide, sactipeptide, prochlorosin, propionicin_SM1, putative bacteriocin, or zoocin_A (Data Set S4). From the Control group, analysis of the 36 MAGs identified 35 pbGCs, represented by 2 BlpUs, 1 enterolysin, 1 lanthipeptide, 1 LAP, 2 lasso_peptides, 22 sactipeptides, 2 prochlorosins, 1 propionicin, and 3 zoocin_A peptides. Nine of the MAGs contained multiple pbGCs, ranging from two to four. Annotation of the MAGs suggested that they likely come from organisms such as *Limosolactobacillus reuteri*, *Streptococcus hyointestinalis*, *Butyricicoccus porcurum*, and from bacteria only predicted at the genus level, such as *Ruminococcus, Faecalibacterium, Prevotella, and Butyricicoccus* (Data Set S4). From the *Salmonella* group, where only 10 MAGs were assembled, our analysis for pbGCs yielded seven candidates that were composed of six sactipeptides and one zoocin_A. The MAGs were predicted as derived from the genera *Velocimicrobium, Prevotella, Campylobacter*, *Helicobacter*, *Alloprevotella*, and *Acidominococcus* (Data Set S4). Sixty-five MAGs were assembled from the *Salmonella* + DFM group. Overall, 92 pbGCs were found in the MAGs from this group, including 73 putative sactipeptides, 5 zoocin_As, 4 lasso-peptides, 3 bovicin_255_peptides, 2 BlpUs, 2 bottromycins, 2 putative bacteriocins, and 1 lanthipeptide. From these pbGC-encoding genomes, MAGs tagged at the genus level included *Lachnospira, Butyricicoccus, Megasphaera, Ruminococcus, Alloprevotella,* and *Dorea,* and at the species level included *Acidaminococcus fermentans*, *Agathobaculum butyriciproducens*, *Holdemanella porci*, and *Streptococcus hyointestinalis*. Several MAGs contained multiple pbGCs, including a member of the Clostridiales (179_maxbinout082.fa) harboring five pbGCs, constituted of four sactipeptides and one bacteriocin, and MAGs from a *Limisoma* sp*., Phascolarctobacterium succinatutens*, and *Desulfovibrio* sp., each containing four pbGCs. In all MAGs with multiple pbGCs, almost all the clusters were for the production of sactipeptides (Data Set S4). Below, we provide insights into the genomic contexts of some of the pbGCs, that is, the zoocin_As, the BlpUs, and the sactipeptides.

### Genomic contexts of the BlpUs, zoocin_As, and sactipeptides from the pig cecum

**Fig 5:**
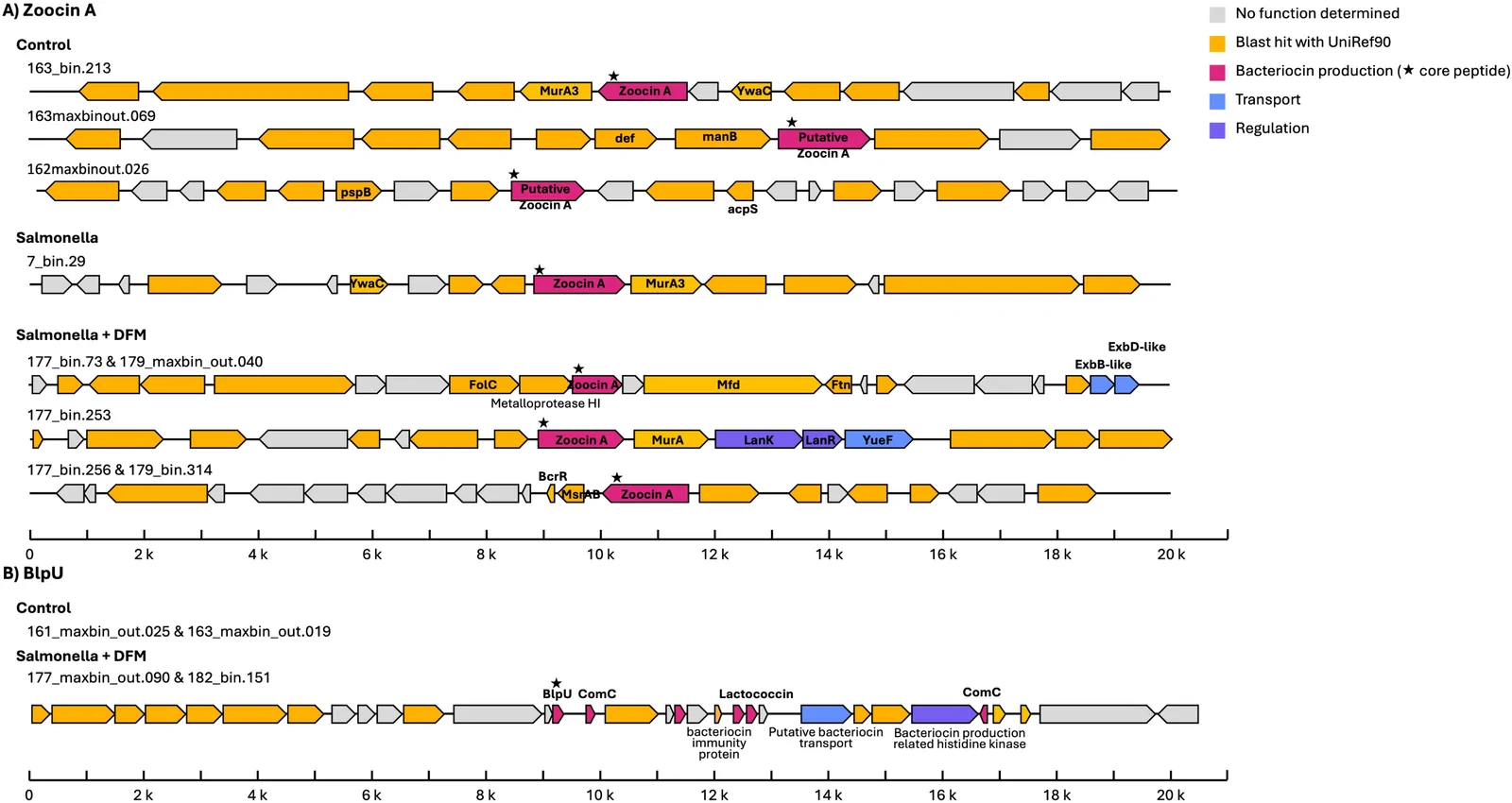
Gene arrangements of zoocin_As and BlpUs in MAGs. Genetic arrangement of (**A**) Zoocin A and (**B**) BlpU clusters in MAGs from Control, *Salmonella,* and *Salmonella* + DFM samples. Bacteriocin clusters were annotated using the BAGEL4 tool, and open reading frames (ORFs) were searched with BLAST against UniRef90 for functional identification. ORFs related to bacteriocin production, transport, and regulation are color-coded, and core peptides for bacteriocin production are marked with a star (✶). The figure focuses on bacteriocin clusters, including nine Zoocin A clusters and four BlpU clusters. MAGs were assigned to the following bacterial groups based on GTDBTk: 163_bin.213.fa, 7_bin.29.fa, 177_bin.256.fa, and 179_bin.314.fa, Lachnospiraceae; 163_maxbin_out.069.fa, Cryptobacteroides sp004552965; 162_maxbin_out.026.fa, UMGS363; 177_bin.73.fa and 179_maxbin_out.040.fa, Helicobacter; 177_bin.253.fa, Lachnospira; and 161_maxbin_out.025.fa, 163_maxbin_out.019.fa, 177_maxbin_out.090.fa, and 182_bin.151.fa, *Streptococcus hyointestinalis*. MAGs assigned to the same bacterial taxon exhibited similar genetic arrangements around the core bacteriocin peptides, indicating conserved gene organization within these clusters.

The zoocins are antimicrobial peptides produced by both gram-positive and gram-negative bacteria (17) and kill or inhibit related or unrelated bacteria (18). Three zoocin_A clusters were found in three different MAGs (163_bin.213—Clostridiales; 163maxbinout.069 —Cryptobacteroides sp; and 162maxbinout.026—Clostridiales) from the Control group, and the genomic contexts suggested they derive from different organisms (Fig. 5A). Two of the MAGs were predicted to come from a member of the Ruminococcaceae (162maxbinout.026—Clostridiales) and the Lachnospiraceae (163_bin.213—Clostridiales), respectively, whereas the third was matched to a *Cryptobacteroides* sp. (163maxbinout.069). Only one zoocin_A cluster was found in the 10 MAGs from the *Salmonella* group, and this MAG (7_bin.29) belonged to a member of the Lachnospiraceae. The genomic context of this zoocin_A did not match any of the three found in the Control group (Fig. 5A). The MAGs from the *Salmonella* + DFM group yielded five zoocin_A genes in five different MAGs (Fig. 5A). The genomic context of the zoocin_A genes in two of the MAGs (177Bin.256 and 179bin.314) was an exact match and suggested they originated from either the same organism or related organisms. In line with this observation, both MAGs were predicted to come from a member of the Lachnospiraceae. Two other MAGs (177bin.73 and 179maxbin.out.040) presented zoocin_A genes that also shared similar genomic contexts, except for a single gene for a peptide (uncharacterized protein MJ0304) that appears to be missing in the latter MAG. Thus, these two MAGs also appear to come from related organisms and were predicted to come from *Helicobacter* sp. 002287135. The fifth MAG (177bin.253) from this group exhibited a genomic context that was different from the other zoocin_A genes found in this experimental group, and the MAG containing this gene was predicted as a member of the genus *Lachnospira*.

Different strains of *Streptococcus thermophilus* have been reported to produce antimicrobial peptides named bacteriocin-like peptide U or BlpUs (19). Four BlpUs were found in our analyses, one from each of two MAGs from the Control group and one from each of two MAGs assembled from the *Salmonella* + DFM group. No BlpU was found in the *Salmonella* group. The genomic contexts of the regions containing the BlpUs were highly conserved in the MAGs, which were predicted as genomes of *Streptococcus hyointestinalis*. The region was characterized by a gene cluster encoding two ComC proteins and at least three other bacteriocins, that is, one Blp family class II bacteriocin and two lactococcins (Fig. 5B).

**Fig 6:**
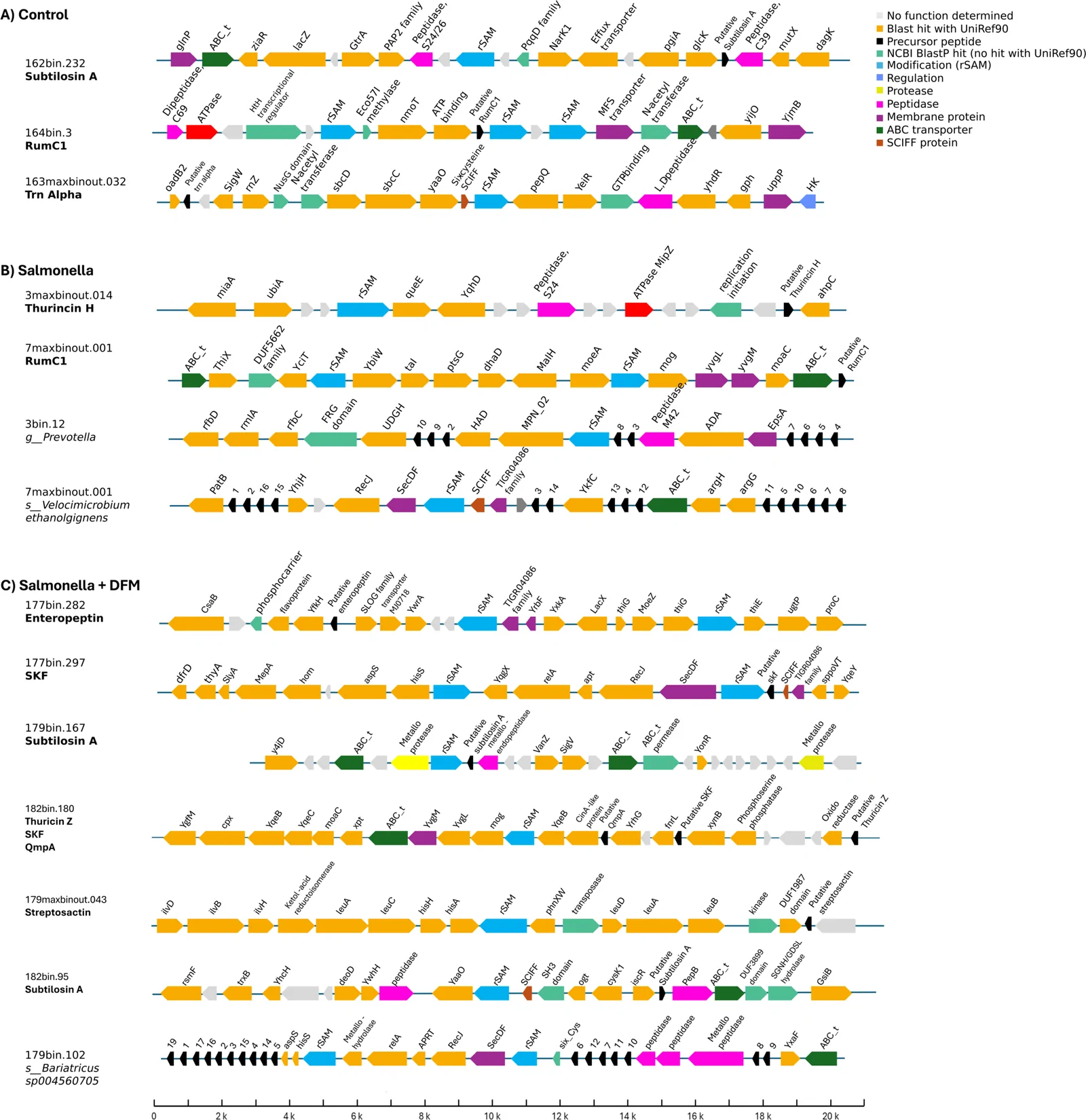
Gene arrangements of sactipeptides in MAGs. Genetic arrangement of sactipeptide gene clusters in MAGs from (**A**) Control, (**B**) *Salmonella* group, and (**C**) *Salmonella* + DFM group. Sactipeptide clusters were annotated using the BAGEL4 tool, and ORFs were searched using BLAST against UniRef90 for functional identification. Putative sactipeptide ORFs are shown in black, while related rSAM proteins are in blue. ORFs related to bacteriocin production, transport, and regulation are also color-coded. The figure highlights a subset of the identified sactipeptide clusters. In total, 101 putative sactipeptide clusters were delineated: 22 in the Control, 6 in the *Salmonella* group, and 73 in the *Salmonella* + DFM group.

The sulfur-to-alpha carbon thioether cross-linked peptides (sactipeptides) belong to RiPPs that show various biological functions, including antibacterial activities (20). Our analyses yielded 101 putative sactipeptides (Data Set S4), with 22 found in the Control group, 6 in the *Salmonella* group, and 73 in the *Salmonella* + DFM group. In Fig. 6, we present a detailed analysis of 3, 4, and 7 putative sactipeptide clusters from the Control, the *Salmonella*, and the *Salmonella* + DFM groups, respectively. The three clusters from the Control group harbored putative subtilosin A, ruminococcin C1, and Trn-αin MAGs designated *Faecousia* sp., *Agathobacter* sp., and Gemmiger sp., respectively (Fig. 6A). The *Faecosia* cluster contained genes encoding one rSAM, two peptidases, an ABC transporter, a membrane protein, and a subtilosin A-like peptide. The *Agathobacter* sp. cluster encoded three rSAMs, a peptidase, an ABC transporter, two membrane proteins, and a ruminococcin C1-like peptide, while the *Gemmiger* sp. cluster harbored one rSAM, a peptidase, no clear ABC transporter but a membrane protein (uppP), a putative trn-α, and a putative six-cysteine forty residue (SCIFF) peptide, representing a potential ranthipeptide or sactipeptide.

In Fig. 6B, we present the genomic contexts of some of the sactipeptide clusters identified in the *Salmonella* group. Associated with the four clusters are a putative thurincin H, ruminococcin C1, several unidentifiable peptides, and a SCIFF peptide, respectively. The thurincin H was in a cluster, from a *Campylobacter hyointestinalis* MAG, that contained also an rSAM, a peptidase, and neither an obvious ABC transporter nor a membrane protein. The ruminococcin C1 was in a cluster in a *Velocimicrobium ethanolgignens* MAG that, in addition, contained genes encoding two rSAMs, two ABC transporters, two membrane proteins, and no obvious peptidase. A *Prevotella* sp. designated MAG had no obvious sactipeptide and harbored an rSAM, a peptidase, no obvious ABC transporter but a membrane protein, and at least nine uncharacterized peptides. The final cluster from this group, also from a *V. ethanolgignens* MAG, contained one rSAM, an ABC transporter, two membrane proteins, a SCIFF peptide, and at least 15 uncharacterized peptides.

From the MAGs assembled for the *Salmonella* + DFM group, 62 putative sactipeptide clusters were delineated. In Fig. 6C, we present the genomic contexts of 7 of these clusters harboring putative enteropeptin, sporulation killing factor (skf), subtilosin A, thurincin Z/putative skf/putative QmpA, streptosactin, subtilosin A, and a cluster with no obvious sactipeptide but at least 17 uncharacterized peptides, respectively. The enteropeptin-containing *Butyricicoccus* sp. MAG harbored genes encoding two rSAMs, two membrane proteins, and neither a clear ABC transporter nor a clear peptidase. The skf-containing *Dorea longicatena* MAG also harbored genes encoding two rSAMs, two membrane proteins, and a putative SCIFF peptide. The subtilosin A-containing *Billifractor* sp. MAG contained genes encoding one rSAM, one peptidase, two proteases, and two ABC transporters. The putative thuricin Z/skf/QmpA or multi-sactipeptide-containing MAG was assigned to a *Dorea* sp. and harbored also in the cluster genes for one rSAM, one ABC transporter, one membrane protein, but no obvious peptidase. A MAG predicted only to come from a bacterium had a cluster encoding a streptosactin and a rSAM, and no other sactipeptide processing-related gene could be identified in the cluster. The subtilosin A-encoding gene cluster in a *Ventricola* sp. MAG also harbored genes encoding one rSAM, two peptidases, one ABC transporter, and a putative SCIFF peptide. The last gene cluster presented from the *Salmonella* + DFM group was in a *Bariatricus* sp. MAG that is also encoded in the cluster 2 rSAMs, 2 peptidases, 1 protease, ABC transporter, a membrane protein, a SCIFF peptide, and at least 17 other peptides.

To gain clearer insight into the sactipeptide-related gene clusters, we focused on the potential rSAM polypeptides identified across our data set. We, thus, examined them for the presence of TIGRFAM domains and the presence of conserved rSAM-associated motifs. As summarized in Table S5, TIGR04068 (a Cys-rich peptide radical SAM maturase) was found in 54.6% of the rSAM proteins and consistently aligned with a conserved GGEPL motif as reported previously (21, 22). Other frequently observed domains included TIGR04085 (radical SAM 4Fe4S, 57.3%) and TIGR04115 (a peptide maturation domain, 44.6%), while TIGR04066 was notably absent. Motif analysis further revealed the near-universal presence of the canonical rSAM (CXXXCXXC) N-terminal binding motif (92.7%) and the SPASM domain (CXXCXXXXXC) in 56.4% of the rSAM proteins. Additionally, a putative SAM-binding motif (TNG) was conserved in 65.5%, and an SXDG motif of unknown function appeared in 47.3% of the rSAM proteins (Table S5). These findings support the functional potential of the predicted sactipeptides and reinforce the diversity of rSAM maturases in the gut microbiomes of pigs from the different groups.

### Predicted structures of the pig microbiome zoocin_As, BlpU, and sactipeptides

**Fig 7:**
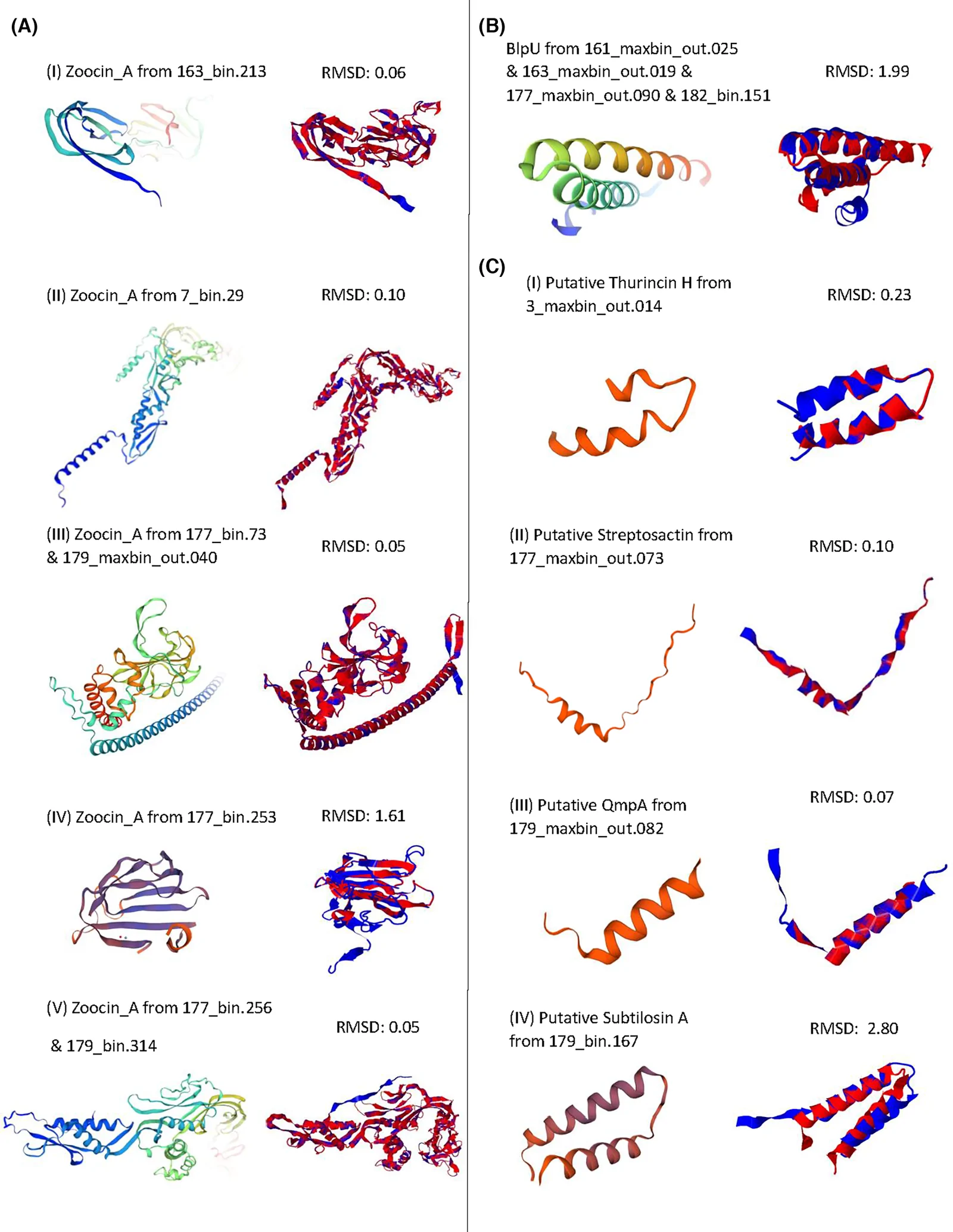
Structural homology. The left side of the figure displays the predicted structures of putative homologs from the current study: (**A**) Zoocin_A, (**B**) BlpU, and (**C**) Sactipeptides. The right side shows the predicted structures (in red) superimposed on reference template structures (in blue), generated using TM-align software. RMSD (root mean square deviation) values are provided for structural comparison. For (**A**) Zoocin_A, the following templates from the AlphaFold database were used for modeling: (I) A0A7 X 7RQT2, (II) A0A316LW00, (III) A0A3D8INY2, and (V) R5K9V1. For Zoocin_A (IV), a published NMR structure was used: 5kvp.1. For (**B**) BlpU, A0A380KSD3 from the AlphaFold database was used as a template. For (**C**) Sactipeptides, a published NMR structure (2LBZ) was used for (I), and the following templates from the AlphaFold database were used for (II–IV): (II) Q0KH46*, (III) A0A116S0I2*, and (IV) O07623. *Annotated as “Uncharacterized protein,” but is a 100% sequence identity match to the published sequence.

To further analyze the putative antimicrobials identified through our analyses, we used computational approaches to obtain their predicted three-dimensional structures. In Fig. 7A, predicted structures of zoocin_As from this study are on the left, and on the right, these structures are superimposed on structures in the database. All the database-derived structures were based on alpha-fold predictions, except for (IV), which is from NMR resolution. As noted above, the BlpUs found in both the Control and the *Salmonella* + DFM groups originated from the same organism, and using the amino acid sequence, we obtained a structure made up of two prominent α-helices (Fig. 7B). A search in the AlphaFold Protein Structure Database yielded a high similarity to a predicted BlpU structure from *Streptococcus infantarius* (A0A380KSD3). The RMSD of the MAG-derived BlpU and the *S. infantarius* structures was 1.99 Å, and with the exception of a short helix that is missing in the predicted structure of the BlpU from the MAG, the two structures were almost a match. In Fig. 7C, we modeled the sequences from four different putative sactipeptides, that is, a thurincin H, a streptosactin, a QmpA, and a subtilosin A, on predicted structures of their counterparts retrieved from the α-fold database. All four predicted structures were made up of α-helices, and their superimposition on their templates exhibited only small deviations. Importantly, the putative thurincin H and the subtilosin A from the MAGs showed a similar structure disposition to the BlpU structure of a helix-turn-helix (Fig. 7B and C). Thus, all predicted structures obtained were similar to others already in the currently available databases, with the RMSD ranging from 0.05 to 1.61 Å.

## DISCUSSION

The use of antibiotics as growth promoters in pork production faces many challenges due to their potential for promoting antibiotic resistance (23–25). Direct-fed microbials that lead to growth promotion without the risks associated with antibiotics are, therefore, receiving attention as alternatives to antibiotics (26–28). The present study was designed to investigate the effect of a *Bacillus*-based DFM supplementation on two disease models common in pigs and to gain insights into the underlying mechanisms. Initial analysis suggested that the 16S rRNA gene sequencing was at enough depth to capture the bacterial community in the ceca of the various groups, allowing us to compare the results.

As observed elsewhere in different breeds of pigs, the bacterial community in the Control group was dominated by Bacillota and Bacteroidota (29), and infection with *Salmonella* or *Salmonella*/PRRSV led to increases in Pseudomonadota and Campylobacterota. Shifts elicited in the pig microbiota by *Salmonella enterica* strains are well documented (30, 31) and have been attributed to the cleansing effect of diarrhea development and expression of genes that endow *S. enterica* strains with a competitive advantage compared with other members of the pig microbiota (31). Importantly, administration of the infection-associated groups of pigs with the DFM, in general, shifted the microbial community at the phylum level toward that of the Control group. Furthermore, by using PCoA analysis, we observed that both challenge models shifted the microbial community away from that of the Control group. However, the DFM application in the *Salmonella* + DFM group homogenized the microbial community (as in the Control group) while at the same time shifting the community towards the Control group. Thus, the results from the PCoA approach mimicked those gleaned from Fig. 1A at the phylum level. Despite the low *Bacillus* dose, the DFM likely influenced the gut ecosystem through metabolite and quorum-sensing effects rather than direct colonization. These inferences align with metagenomic findings but require validation using transcriptomic, metabolomic, and culture-based approaches.

Several reports have demonstrated that gut microbiota dysbiosis can impact lung physiology (32–34). Based on these observations and our own reports (35, 36), we examined the pig lung to determine whether the shift in the gut microbial community translated to the physiological state of the lungs. *Salmonella* infection resulted in hepatization of the lung, and significantly, the presence of the DFM led to a healthier lung histology than that of the Control group. The application of the DFM to the *Salmonella* + PRRSV group, on the other hand, did not significantly impact the lung histology compared to the absence of the DFM. Analysis of the cecal samples from the pigs infected with *Salmonella* showed a community characterized by higher numbers of bacteria that included members of *Ruminobacter*, *Anaerobiospirillum*, and *Campylobacter*. DFM application then led to a higher presence of organisms such as *Clostridium sensu stricto 1, Actinobacillus, Mucispirillum, Shuttleworthia, Oxalobacter,* and *Romboutsia*. Increases in *Campylobacter* in the pig gut microbiota upon challenge with a strain of *Salmonella enterica* have also been reported by others (30). For the pigs with the double infection, bacteria such as members of *Odoribacter*, *Treponema, Tyzzerella,* and *Tuzzerella* predominated in the absence of DFM, while DFM supplementation led to increases in genera such as *Romboutsia* and *Terrisporobacter.* A number of bacterial genera, including *Odoribacte*r and *Anaerospirillum*, found in the single and double infections without DFM have been reported to be associated with diarrhea and inflammation in pigs (37–39). Thus, infection with *S. enterica* serotype Choleraesuis led to alteration in the pig cecal microbiota, an observation that has been reported for other *Salmonella enterica* serovars in pigs (30) and other animal models such as the chick and chicken (40, 41) and the mouse (42). Our results, therefore, demonstrated that DFM application shifts the bacterial population in both the single- and dual-disease pig models.

The above analyses were based on 16S rRNA gene sequence data, which only informed us of bacterial community dynamics. Thus, we hypothesized that examining the microbiome would provide a deeper insight into the changes leading to the differences in the lung histology of the pigs. To probe our hypothesis, we reconstructed bacterial genomes from our metagenomic data by generating MAGs. The analyses of the MAGs showed that the majority in the *Salmonella* + DFM and the Control groups belonged to the Bacillota, while 50% of the MAGs from the *Salmonella* group were from the Bacteroidota. Due to the smaller number of MAGs generated from the *Salmonella* group, we concluded that comparisons among the groups will not be optimal; however, the insights gained from the microbiomes (the genetic contents) could inform the changes resulting in the differences in the lung histology and therefore allow future research to build on this data. Importantly, we attributed the dominance of the host DNA in the genetic material extracted from the *Salmonella* group, in part, to diarrhea induced by the infection, as reported elsewhere (30). Thus, although the dominance of the eukaryotic or host DNA in some of our samples still allowed 16S rRNA gene amplification (as the primers are target or bacterial-specific), it limited access to bacterial DNA in the metagenomic sequencing.

We observed many important similarities between the Control and the *Salmonella* + DFM groups, including the predominance of MAGs assigned to the *Streptococcus*, *Ruminococcus*, and *Eubacterium* in each group. Bacteria have evolved a wide range of weapons for outcompeting their competitors in a shared environment, and among these are antibiotics and toxic proteins (43). Our analyses yielded a strong similarity in the antibiotic resistance genes present in the Control and the *Salmonella* + DFM groups. The virulence factors, on the other hand, were far higher in number in the *Salmonella* + DFM group compared to the Control and the *Salmonella* groups. Bacterial quorum-sensing is key to cell/cell communication and is further known to be harnessed for controlling expression of virulence factors and antibiotic resistance genes (44). Thus, quorum-sensing is currently seen as a potential target alternative to antibiotics for mitigating the increase in antibiotic resistance (44). These findings may suggest that the *Salmonella* + DFM and Control groups’ microbiomes retained strong competitive exclusion capacity. The prevalence of cyclic lactone-autoinducers, which function in quorum-sensing, showed an incidence of 1 in 10 MAGs for the *Salmonella* group and 1 in 2 MAGs in the *Salmonella* + DFM and Control groups. Hence, similar to the observation with the antibiotic resistance genes and virulence factors, these key players in bacterial warfare were enriched in the *Salmonella* + DFM and Control groups. Importantly, recent evidence has shown that *Bacillus*-based DFMs can disrupt quorum-sensing in pathogenic bacteria, leading to decreases in the virulence strategies of the pathogenic organism (45–47). Thus, it could be that the DFM in the present study directly or indirectly induced the production of cyclic-lactone-autoinducers as a means to disrupt *Salmonella* quorum-sensing signaling, leading to the latter pathogen being outcompeted in the presence of the DFM.

The influence of SCFAs in lung health has been documented through a mechanistic explanation of the gut–bone marrow–lung axis (48). Here, gut microbiota and gut-derived SCFAs regulate hematopoiesis to prime the immune system against infections and allergenic responses in the periphery (48, 49). In the present study, there were generally higher numbers of MAGs with complete pathways for acetate and butyrate production in the *Salmonella* + DFM groups than the Control group, which also had higher numbers compared to the *Salmonella* group. The large number of incomplete pathways for propionate may reflect an inherent complexity of the propionate pathway or limited information on the pathways in the current databases, that is, the potential to have missed some key enzymes involved in propionate production. Nonetheless, the higher numbers of both partial and full propionate pathways and also acetate and butyrate pathways in the *Salmonella* + DFM and Control groups suggest that these two groups harbored more robust microbial populations with potential for SCFA production and therefore more benefits from these important metabolites.

Intestinal bacteria produce antibacterial molecules known as bacteriocins with the capacity to eliminate other bacteria, and the development of metagenome-based screens has expanded insight into the abundance of bacteriocin biosynthetic gene clusters encoded by different bacteria (50). Our analyses of putative bacteriocins in the MAGs yielded diverse types of these important antimicrobials, including zoocins, BlpUs, and lactococcins. However, our results showed dominance in our MAGs of putative sactipeptides, a new source of peptides that have functions including antibiotics. Zoocins include lactocin, enterocin, and zoocin A, the latter protein from *Streptococcus equi* subspecies zooepidemicus being a zinc-metallopeptidase reported as a 285 amino acid pre-peptide that is processed, through cleavage of the leader sequence, to an active polypeptide of 262 amino acids (18, 51, 52). The BlpU gene clusters in the present work were associated with a MAG designated *Streptococcus hyointestinalis*, and in a related bacterium, *Streptococcus mutan*s, competence and bacteriocin production are controlled by the ComC and the ComED two-component signal transduction system (53). Consistent with this observation, our analyses disclosed that the BlpU gene cluster in the *S. hyointestinalis*-designated MAG includes two ComC-like peptides encoding genes, as well as genes encoding a ComD-like histidine kinase and a ComE-like response regulator. The cluster also encodes other proteins of significance to bacteriocin function, including a bacteriocin transport accessory protein and a bacteriocin immunity protein, the latter likely functioning to protect *S. hyointestinalis*. Furthermore, the association of the gene cluster with transposase-related genes suggests that the cluster was acquired or may be transferable to other bacteria. Also, importantly, the genomic contexts of the BlpUs in the two MAGs designated *S. hyointestinalis* in the Control were the same as the two found in the *Salmonella* + DFM group, suggesting that the same organism or similar strains were present in both the Control and *Salmonella* + DFM groups.

Several potential sactipeptides were delineated in the MAGs from the present study. The sactipeptides are currently considered potential alternatives to conventional antibiotics (54). They are members of the RiPPs and are classified as class I bacteriocins (50). The most significant characteristic of the sactipeptides is an intramolecular thioester bond that cross-links the sulfur atom of a cysteine to the α-carbon atom of an acceptor amino acid in the peptide, a reaction catalyzed by a radical S-adenosylmethionine (rSAM) enzyme (20). Other genes encoded in the cluster with the sactipeptide and the rSAM genes include those for protease, membrane protein/transport systems, regulatory factors, and thioredoxin activities (20, 55). Earlier examples of sactipeptides, in the order of their discoveries, include subtilosin A, a secondary metabolite previously shown to disrupt quorum-sensing in *Salmonella enterica* (47), thurincin H, skf, the two-component thurincin CD (Trn-α and Trn-β), ruminococcin C1, and streptosactin (20). Importantly, in the present study, 1 and 7 putative sactipeptide clusters from the Control and the *Salmonella* + DFM group, respectively, had no identifiable rSAM encoding genes. Thus, based on the critical function of the rSAM enzyme, we revised the number of putative sactipeptides in the Control and the *Salmonella* + DFM groups to 21 and 66, respectively. We recognize, however, that some of the sactipeptide BGCs have several peptide- and protein-encoding genes with unknown function, and some of these may be involved in the process leading to the maturation of the putative bacteriocins/sactipeptides. Thus, their discovery in the present study may facilitate future biochemical and genetic characterizations to expand knowledge on these new antimicrobial candidates, and this may include expression of the recombinant gene products for antimicrobial activity assays.

As noted above, there were several cases where multiple peptides associated with the sactipeptide clusters could not be assigned a function. Thus, we hypothesize that some of these peptides are novel sactipeptides. Also importantly, some MAGs encoded multiple bacteriocins of likely different activities; for example, a *Faecalibacterium*-like MAG contained genes coding for putative lanthipeptide, sactipeptide, and prochlorosin activities, respectively, while others encoded multiple forms of potentially similar activities. Metagenomic data from intestinal *Bacteroides* spp. (56) and rumen bacteria (21) suggested that they are sactipeptide producers (50), and importantly, a thurincin CD and ruminococcin C from *Bacillus thuringiensis* and *Ruminococcus gnavus*, respectively, have been demonstrated as being highly active against *Clostridiodes difficile* (57, 58), a pathogen of significant health consequence (59). Furthermore, a sactipeptide named SacP3 from *Paenibacillus polymyxa* #23 has been shown to exhibit antimicrobial activity against several gram-positive bacteria, including multi-drug resistant *Staphylococcus aureus* (60). Thus, the high numbers of sactipeptides in the *Salmonella* + DFM group suggest enhanced antimicrobial activity that might have been inhibitory to the *Salmonella* and other potential pathogens in the pig, leading to a significantly healthier lung.

The domesticated pig is a major source of protein for an ever-expanding human population, while also serving as an animal model for biomedical research (61, 62). Antibiotic use has been integral to the commercial production of pigs, as growth promoters and suppressors of disease (63). The potential for the spread of antibiotic resistance by this significant agricultural sector has, however, resulted in efforts to find alternatives to replace antibiotics. DFM that remodels the native microbiota to a more resilient community represents a potential replacement for sub-therapeutic antibiotic administration in pig production. Here, we demonstrate that a *Bacillus*-based DFM remodels the pig gut microbiota to one that wards off *Salmonella* infection, a common occurrence in commercial pig farms. Our molecular analysis suggested a shift of the microbiota to a community enriched in the capacity to produce naturally occurring antimicrobials and immune-stimulating SCFA.

It is important to note, however, that the present study relies mostly on genomics, computational, and bioinformatic analyses to assess microbial community dynamics to provide insights into the mechanisms underlying the function of the DFM under investigation. Nonetheless, the approaches also offered critical insights into the genetic potential associated with the shifts in taxa (or the microbiomes), focusing especially on novel antimicrobial peptides. While their direct functional validation remains, we posit that by uncovering these potentially novel antimicrobial peptides, this study offers the opportunity for their detailed biochemical characterization to unravel their bioactivities and physiological roles. It is anticipated that further studies will pave the way for the application of the peptides in both animal agriculture and human health. We further note that the small group size (*n*=4 per treatment) likely reduced statistical power for co-infection comparisons. This number was based on the feasibility of collecting detailed microbiome and histopathological data from each animal. Sample loss due to high host DNA contamination further limited metagenomic comparisons. Furthermore, the absence of a DFM-only group limits the interpretation of probiotic effects under non-infectious conditions. Future studies with larger cohorts, defined power analyses, and inclusion of unchallenged DFM controls will be important to confirm and extend our findings.

## MATERIALS AND METHODS

### Animals, diet, housing, and experimental design

The research aimed at evaluating the impact of a DFM cocktail on the gastrointestinal microbial community composition and parameters of lung health in pigs challenged with either *Salmonella enterica* serotype Choleraesuis (Sc) alone or in combination with PRRSV. The animals used in the study were 5-week-old mixed-breed pigs obtained from a research farm at U of I, weaned on the same day (Table S6). Thus, the pigs were sourced from a high-health research facility and confirmed free of PRRSV, *Mycoplasma hyopneumoniae*, and *Salmonella* enterica prior to study initiation. Baseline screening included fecal swabs and qPCR performed at the University of Illinois Veterinary Diagnostic Laboratory (VDL), which confirmed all animals were negative for these pathogens and clinically healthy before experimental challenge. Animals were housed in a biosafety level 2 (BSL2) biocontainment facility at U of I, and each experimental group was housed in a separate, environmentally isolated pen. Group 1 was a control, where four pigs were fed neither DFM nor challenged with either Sc or PRRSV. In Group 2, four pigs were challenged with Sc but not fed DFM. In Group 3, four pigs were fed Sc and administered DFM. In Group 4, four pigs were administered both Sc and PRRSV but not DFM, and in Group 5, four pigs were fed PRRSV and Sc and administered DFM. The DFM consisted of Provent ECL (lot CX0020180321), a microbial cocktail comprised of six *Bacillus* strains (S10-48, SB0-48, S10-44, S10-49, S10-50, and S10-51) applied at 3 lb/ton (~1.1 × 10^6^ CFU/g) of feed and fed as dry spores with a carrier comprised of calcium carbonate, maltodextrin, and mineral oil. The DFM cocktail was developed by Microbial Discovery Group (MDG, Oak Creek, WI, USA). Pigs began their designated diets (Table S7) with or without DFM 3 weeks prior to administration of the Sc challenge (day −21). On day 0, animals in Groups 2, 3, 4, and 5 were challenged orally with 10^9^ CFU of *Salmonella enterica* serovar Choleraesuis strain 4271GB, a Kunzendorf variant belonging to the *Salmonella* O antigen C1 serogroup (Choleraesuis). Challenge was performed by administering a pill containing gelatin capsules loaded with the Sc into the esophagus. Pigs in Group 1 received a gelatin capsule with a mock inoculum. Three days later (day 3), animals in Groups 4 and 5 were intranasally challenged with 5 × 10^4^ TCID50 of PRRSV strain NADC20 (P), and pigs in Groups 1, 2, and 3 received a mock virus inoculum. Table S1 summarizes the experimental design. Animals were observed for 8–9 days post-bacterial challenge for clinical outcomes.

### Sample collection

Animals were observed for 8–9 days post-bacterial challenge for clinical outcomes. After euthanasia and necropsy, lung tissue, gut mucosa, and digesta of the cecum section of each pig were snap-frozen in liquid nitrogen and stored at −80°C until DNA extraction. For digesta, luminal material was gently collected, after which the exposed mucosa was flushed with sterile 0.1% peptone to remove any visible digesta, and a sterile cell scraper was used to scrape along the surface of the exposed tissue, collecting a mucosa sample of approximately 100–200 µg.

### Genomic DNA extraction

Community genomic DNA was extracted using DNeasy Powerlyzer Powersoil (Qiagen, the Netherlands) according to the manufacturer’s instructions. DNA quality was assessed using a NanoDrop spectrophotometer (260 nm/280 nm; ThermoFisher Scientific, USA) and by electrophoresis on 1% agarose gel. DNA storage prior to analysis was at −20°C.

### Full-length 16S rRNA gene amplification and PacBio sequencing of gut samples

Full-length 16S rRNA gene amplicons were generated from the community genomic DNA originating from the gut of the pigs with the barcoded full-length 16S rRNA gene primers from PacBio (United States) and the 2× KAPA HiFi Hot Start Ready Mix (Roche, United States). The forward and reverse primers used for the PCR amplifications were 5′-AGRGTTYGATYMTGGCTCAG-3′ and 3′-RGYTACCTTGTTACGACTT-5′, respectively. Sheared community genomic DNA was converted to a library with the SMRTBell Express Template Prep Kit 2.0 (PacBio, United States). The library was then sequenced on 1 SMRTcell 8M on a PacBio Sequel II using the circular consensus sequencing (CCS) mode and a 10-h movie time. The CCS analysis was done using SMRTLink V8.0 using the following parameters: ccs --min-length 1200 --max-length 2000 --min-passes 3 --min-rq 0.999. lima --ccs --different --split-bam-named. The 16S rRNA gene sequencing was performed at the DNA Service Lab at the University of Illinois Urbana-Champaign.

### Sequencing of bacterial 16S rRNA Shoreline amplicons in lung tissue

To circumvent the high occurrence of pig (host)-derived reads from the lung tissue DNA, Shoreline amplicon sequencing was used to assess the bacterial communities in the lungs of pigs. The phylogenetic amplicons were prepared with the Shoreline Biome StrainID kit (Shoreline Biome, United States). The nucleotide sequences of the forward and reverse primers were 5′-AGRRTTYGATYHTDGYTYAG-3′ and 3′-AGTACYRHRARGGAANGR-5′, respectively. The DNA amplicons were converted to a library with the SMRTBell Express Template Prep kit 2.0. The library was then sequenced on 1 SMRTcell 8M on a PacBio Sequel II using the CCS sequencing mode and a 15-h movie time. The CCS analysis was carried out using SMRTLink V10.0 with the following parameters: ccs --min-passes 3 --min-rq 0.999. Demultiplexing was done using Sbanalyzer (Shoreline Biome, United States), and the data were not adaptor trimmed.

### Metagenome sequencing of gut samples with Oxford Nanopore (long reads)

The community genomic DNA extracted from digesta samples of the cecum of each pig (Table S2) in Groups 1–3 was sequenced using Illumina and Oxford Nanopore Technologies (ONP). The genomic DNA was converted into a Nanopore library with the SQK-LSK109 kits. The library was sequenced on a SpotON R9.4.1 FLO-MIN106 flowcell for 72 h, using a GridIONx5 sequencer (Oxford Nanopore, Oxford, UK). Basecalling was performed with Guppy 5.1.13 (Oxford Nanopore, Oxford, UK) using the High Accuracy Mode. Reads were trimmed of adaptors with Porechop 0.2.3 (https://github.com/rrwick/Porechop).

### Metagenome sequencing of gut samples with Illumina (short reads)

For metagenomics Illumina sequencing, the shotgun libraries were prepared with the Hyper Library construction kit from Kapa Biosystems (Wilmington, MA, USA). After pooling of the libraries, quantification by qPCR was performed. The samples were sequenced on a single SP lane for 151 cycles bidirectionally on a NovaSeq 6000 (Illumina, San Diego, CA). Fastq files were generated and demultiplexed with the bcl2fastq v2.20 Conversion Software (Illumina).

### Bioinformatics analysis of full-length 16S rRNA genes

The 16S rRNA gene sequence analyses were completed by the High-Performance Computing in Biology (HPCBio) at the University of Illinois Urbana-Champaign. The PacBio data targeting the entire 16S region were quality-assessed using FASTQC (64) and then processed using the PacBio-adapted version of the TADA Nextflow-based workflow (https://github.com/h3abionet/TADA/blob/2021-Shoreline-Project/pacbio.nf) that implements the DADA2 workflow (65) for dereplicating and denoising reads to generate amplicon sequence variants (ASVs). Sequences with a minimum of 1,000 nt or less than or equal to 5,000 nt underwent primer sequence removal and minimal quality trimming, after which, only sequences with a maximum expected errors (EE) score of 2, and no unclassified bases were retained. Error estimation further included the additional parameters: “errorEstimationFunction=PacBioErrfun.” Across all samples, the workflow retained 94.77% of total sequences, although retention varied by sample, with the greatest losses occurring during the denoising step. Apart from default settings, denoising used the settings “BAND_SIZE=32, OMEGA_A=1e-10, OMEGA_C=1e-80, PSEUDO_PREVALENCE = 2.” This was followed by default steps that included dereplication into ASVs and taxonomic classification using the DADA2 implementation. The taxonomic classification was done with the RDP Classifier (66) and with the Silva v138 database customized for the DADA2 application of long-read data (67, 68). Multiple sequence alignment of the resulting ASV sequences was performed by DECIPHER (69), subsequently followed by the production of a midpoint-rooted Fasttree (70) phylogenetic analysis to be used in downstream data analysis. Raw counts, taxonomic assignments, and the phylogenetic tree were imported into R (v.4.0.5) (71) using the package phyloseq (v.1.34.0) (72). ASVs were filtered out if they were mitochondrial or chloroplast, did not have an assigned phylum, or were the only ASV in a phylum. The resultant ASVs were used for alpha diversity analyses. Agglomeration was then performed on ASVs that had phylogenetic distances < 0.05, after which prevalence filtering with a threshold of 5% was applied, leaving 897 “taxa” for beta diversity (Bray–Curtis) statistical analyses. Alpha and beta diversity analyses, as well as differential abundance analysis (DESeq2) (73) and custom lists of specific taxa, were performed and extracted using phyloseq and vegan (v.2.5.7) packages. For the beta diversity analyses, relative proportion normalized counts were utilized to perform PERMANOVA and ANOSIM tests using vegan functions “adonis2” and “anosim” to assess the significance of differences in Bray–Curtis distance between control and treatment. The R code used to conduct this analysis can be found at https://github.com/HPCBio/icann-Nov2020-16S.

### Metagenomic analysis of gut samples (long and short reads)

Samples were sanitized by removing host reads using the pig reference genome (https://ftp.ncbi.nlm.nih.gov/genomes/all/GCF/000/003/025/GCF_000003025.6_Sscrofa11.1/GCF_000003025.6_Sscrofa11.1_genomic.fna.gz). Kneaddata, version 0.10.0 (https://huttenhower.sph.harvard.edu/kneaddata/), was used to sanitize Illumina reads, and minimap2, version 2.21-IGB-gcc-8.2.0 (73), was used to sanitize ONP reads. Five samples lost a significant amount of reads because they aligned to the pig reference genome. Namely, samples with IDs 3, 7, 172, 173, and 181 (Table S3). Metagenome assembly with sanitized Illumina reads was performed on all samples with metaSPades (74), version 3.15.3, using the nextflow pipeline (https://github.com/HPCBio/Metagenome-Workflow/tree/dev). Metagenome assembly with sanitized ONP reads was performed with metaFlye (12), (version 2.7), using the nextflow pipeline (https://github.com/HPCBio/Metagenome-Workflow/tree/ONP). Polishing of the ONP metagenome assemblies was performed with NextPolish (11), version 1.4.1, using the sanitized Illumina reads. Evaluation of metagenome quality was assessed before and after the polishing step with MetaQuast (75) (version 5.0.2) and Kraken2 (76) (version 2.1.2). The MetaQuast database used was as follows: SILVA_123_SSURef_Nr99_tax_silva.fasta. The Kraken2 database used was as follows: PlusPF (url: https://benlangmead.github.io/aws-indexes/k2 version 20220908). Binning of the polished ONP metagenome assemblies was performed with three different binning tools: metaBAT2 (77), version 2.15, maxbin2 (78) (version 2.2.7), and DAS_Tool (79) (version 1.1.2). Metagenome bin quality assessment was performed with CheckM (80) (version 1.1.3) and subsequently plotted with Microsoft Excel. Taxonomic assignment of the bins was performed with GTDBTk (81) (version 2.1.1) with the “gtdbtk classify_wf” workflow. The GTDB database used was version R207_v2/release207_v2. Functional annotation of the bins was performed with MetaGeneMark-2 (82) (version 20210406) and with InterProScan (83) (version 5.47-82.0). A list of known pathogens of interest was identified, then retrieved from NCBI (https://www.ncbi.nlm.nih.gov/pathogens) to be compared to the taxonomic results obtained with GTDBTk (Data Set S1). Used commands in metagenomic analysis can be found at https://github.com/HPCBio/icann-Nov2020-16S. For *de novo* phylogenetic tree construction, GTDBTk was applied using the de_novo_wf pipeline (81). Included settings were --skip_gtdb_refs and a c --custom_taxonomy_file, so that the tree is based on only the newly constructed genomes and their taxonomic assignment from GTDBTk. The MAGs were filtered from our bins at 95% completeness and with less than 2% contamination.

### Analyses of MAGs to identify anti-microbial genes

Online databases were used to evaluate the presence of ARGs (https://card.mcmaster.ca/analyze/rgi) and VFs (total hits; http://www.mgc.ac.cn/VFs/main.htm) in the MAGs. ARGs were identified based on strict hits with high-quality coverage, while VFs were detected using the VFDB database and identified through BLASTn. BGC analysis was conducted using antiSMASH version 7.0 with detection strictness parameters set to relaxed mode to capture well-defined clusters containing all required components and partial clusters missing one or more functional components. The analysis included additional antiSMASH features such as KnownClusterBlast, SubClusterBlast, ActiveSiteFinder, RREFinder, and TFBS analysis to provide functional insights into the predicted clusters. To specifically identify bacteriocin-associated gene clusters, BAGEL4 (http://bagel4.molgenrug.nl/) was used, as it is optimized for detecting bacteriocin biosynthesis-related genes. Unlike antiSMASH, which broadly screens for various secondary metabolite biosynthetic pathways, BAGEL4 specializes in identifying bacteriocins and RiPPs. Genes related to potential biosynthetic processes were counted as a single hit per genome locus. MAG labels were manually annotated to reflect phylogenetic assignments derived from GTDBTk.

### Predicting short-chain fatty acid pathways

MAGs were analyzed for the presence or absence of genes related to SCFA production, specifically acetate, propionate, and butyrate. The MAGs were first annotated using Prokka (Version 1.13.4) on an in-house Linux-based platform. The resulting annotation files were uploaded to BlastKOALA (https://www.kegg.jp/blastkoala/) and eggNOG-mapper (http://eggnog-mapper.embl.de/) to identify corresponding KEGG pathways and genes. The specific genes analyzed in this study are outlined in the legend for Excel S2 and Fig. S8). Genes such as butyrate-CoA transferase and propionate CoA-transferase were successfully annotated with Prokka; however, the corresponding KEGG numbers did not appear in the eggNOG or BlastKOALA databases. The identities of the two enzymes were further confirmed by running their respective sequences on BLASTp to ensure the correct annotations. No other enzymes were re-annotated through BLASTp, as the initial Prokka annotation was confirmed by the BlastKOALA and eggNOG-mapper databases. A MAG was considered to encode a specific enzyme if it was annotated in at least one of the databases alongside the Prokka annotation (e.g., Prokka and BlastKOALA, or Prokka and eggNOG). This approach was used because different databases may offer varying annotations. MAGs were then categorized based on the presence, partial presence, or the absence of pathways. These results were further visualized on the phylogenetic tree shown in Fig. 4.

### Identifying putative sactipeptides

After identifying sactipeptide BGCs, short ORFs (25–70 amino acids) within the cluster were run on NCBI BLASTp against a set of 11 published sactipeptide sequences (54). These specific sequences were selected because some of them are not yet annotated in the NCBI BLAST database. As a result, using a targeted set of 11 known sactipeptides helped to improve the sensitivity of the search (Table S8). Sequences that showed a BLASTP similarity score exceeding 25% identity to any of these known sequences were classified as putative sactipeptides for further analysis.

### Predicted structures for sactipeptides

For the putative sactipeptides, which are relatively novel and may lack pre-existing templates in the Swiss-Model database, we first searched the known published sactipeptide sequences (54) in UniProt to determine whether there were any available experimentally determined structures that could serve as templates. For peptides with available experimental nuclear magnetic resonance (NMR) structures, these were used as templates for structural predictions. In the absence of experimentally determined structures, we accessed the AlphaFold-predicted structures (84–86). Specific reference templates are indicated in the figure legend (Fig. 7). The sequences were uploaded to Swiss-Model along with their respective PDB template files (either their experimentally determined structures or AlphaFold models). The “User template” setting in Swiss-Model was used, allowing for more tailored and accurate structure predictions. The resulting predicted 3D structures were downloaded as PDB (Protein Data Bank) files for subsequent analysis and superimposition. This approach allowed us to use AlphaFold-predicted structures within a uniform homology modeling pipeline, ensuring consistent structural refinement and format compatibility across all peptides, whether template-based or *de novo*.

### Predicted structures for Zoocin A and BlpU

To generate predicted three-dimensional structures of the putative Zoocin A and BlpU proteins, their amino acid sequences were submitted to Swiss-Model (https://swissmodel.expasy.org/) using the “Sequence” setting. The tool performed homology modeling by identifying suitable structural templates and generating predicted models based on sequence similarity. For each protein, the predicted structure was generated and downloaded as a PDB file. The highest-ranking hit for each sequence from Swiss-Model was then downloaded and used as the template for subsequent superimposition analysis.

### Superimposition and RMSD calculation

Structural comparison of the predicted peptide or protein structures to their respective templates was performed using TM-Align, a tool for pairwise structural alignment and comparison of protein structures. Both the predicted structures from Swiss-Model and the template structure (either NMR or AlphaFold model) from UniProt were uploaded in PDB format to the TM-Align web server (https://zhanggroup.org/TM-align/). The use of a template was necessary to provide a reference for validating the accuracy of the predicted structures by comparing them to known conformations. TM-Align aligned the two structures and provided an RMSD value (in Ångstroms, Å), representing the average distance between aligned backbone alpha-C atoms. The superimposed structures were visualized and exported as images directly from the TM-Align interface.

### Lung sample staining

Samples from the lung tissues collected were subjected to staining of thin sections and scoring for pathological findings (Table S4). The staining was performed using the Masson’s Trichrome method (87) (Polysciences Inc, United States). The method is applied to characterize tissue status during disease development and was used to assess the effect of the DFM cocktail on the lung tissues. Fixation of frozen samples was performed with neutral buffered saline (NBF) and subsequent manufacturer’s instructions for frozen tissue. After staining, images were acquired on a digital image scanner (Nanozoomer 2.0HT, Hamamatsu, Japan) and cropped using NDP.view2 (Hamamatsu, Japan). Seven experts in the field of pulmonary disease blinded for the treatments scored the lung pathological findings.

## Data Availability

These genomic data have been submitted to the NCBI SRA under BioProject ID PRJNA1279271.

## SUPPLEMENTAL MATERIAL

Data Set S1 aem.00972-26-s0001.xlsx Metadata for phylogenetic tree construction of MAGs.

Data Set S2 aem.00972-26-s0002.xlsx Butyrate, acetate, and propionate production-related genes in microbial genomes in control, *Salmonella* challenge, and DFM groups.

Data Set S3 aem.00972-26-s0003.xlsx Biosynthetic gene clusters in microbial genomes in control, *Salmonella* challenge, and DFM groups.

Data Set S4 aem.00972-26-s0004.xlsx Bacteriocin-related genes in microbial genomes in control, *Salmonella* challenge, and DFM groups.

Data Set S5 aem.00972-26-s0005.xlsx Bacteriocin homologs.

Supplemental material aem.00972-26-s0006.pdf Fig. S1 to S8; Tables S1 to S8.

ASM does not own the copyrights to Supplemental Material that may be linked to, or accessed through, an article. The authors have granted ASM a non-exclusive, world-wide license to publish the Supplemental Material files. Please contact the corresponding author directly for reuse.
