# Supplementary material for: A Bacillus-based direct-fed microbial mixture remodels the gut microbiome to augment the respiratory health of Salmonella-infected pigs
Source: Appl Environ Microbiol. 2026 Jul 10;92(8):e00972-26. doi: 10.1128/aem.00972-26 (PMC13488306; doi:10.1128/aem.00972-26)
Supplement: Supplementary file 1 [file aem.00972-26-s0006.pdf]

Supplementary materials

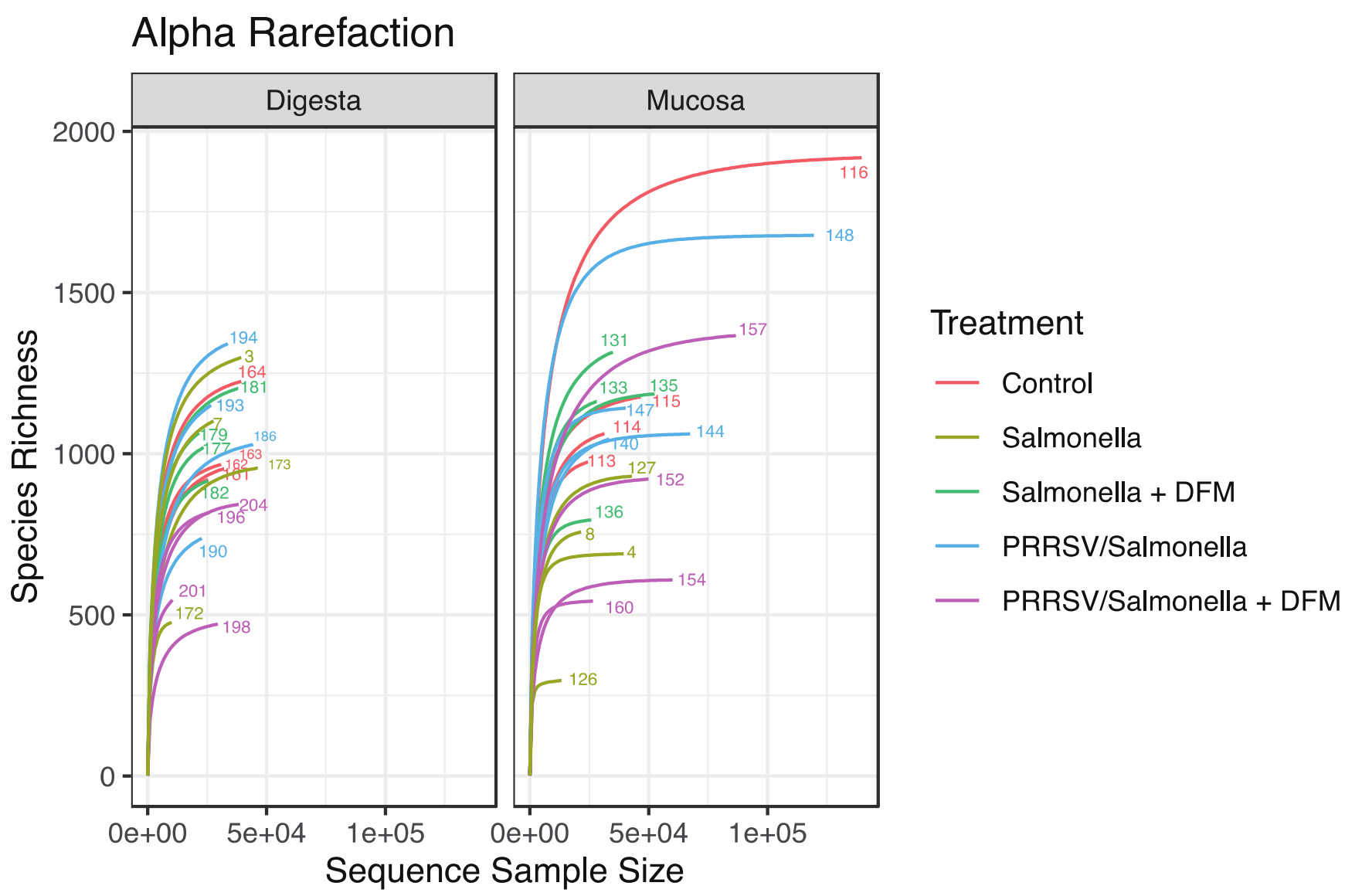

Figure S1. Alpha rarefaction curve for digesta and mucosa samples. Species richness or abundance (Y-axis) is plotted into rarefaction curves that are colored by treatment type and labeled by sample using ranacapa and ggplot packages. Sequence sample size is plotted on the X-axis, with a rarefaction step size of 1000 reads used to draw curves. Most curves plateau indicating that all dominant species have been successfully captured.

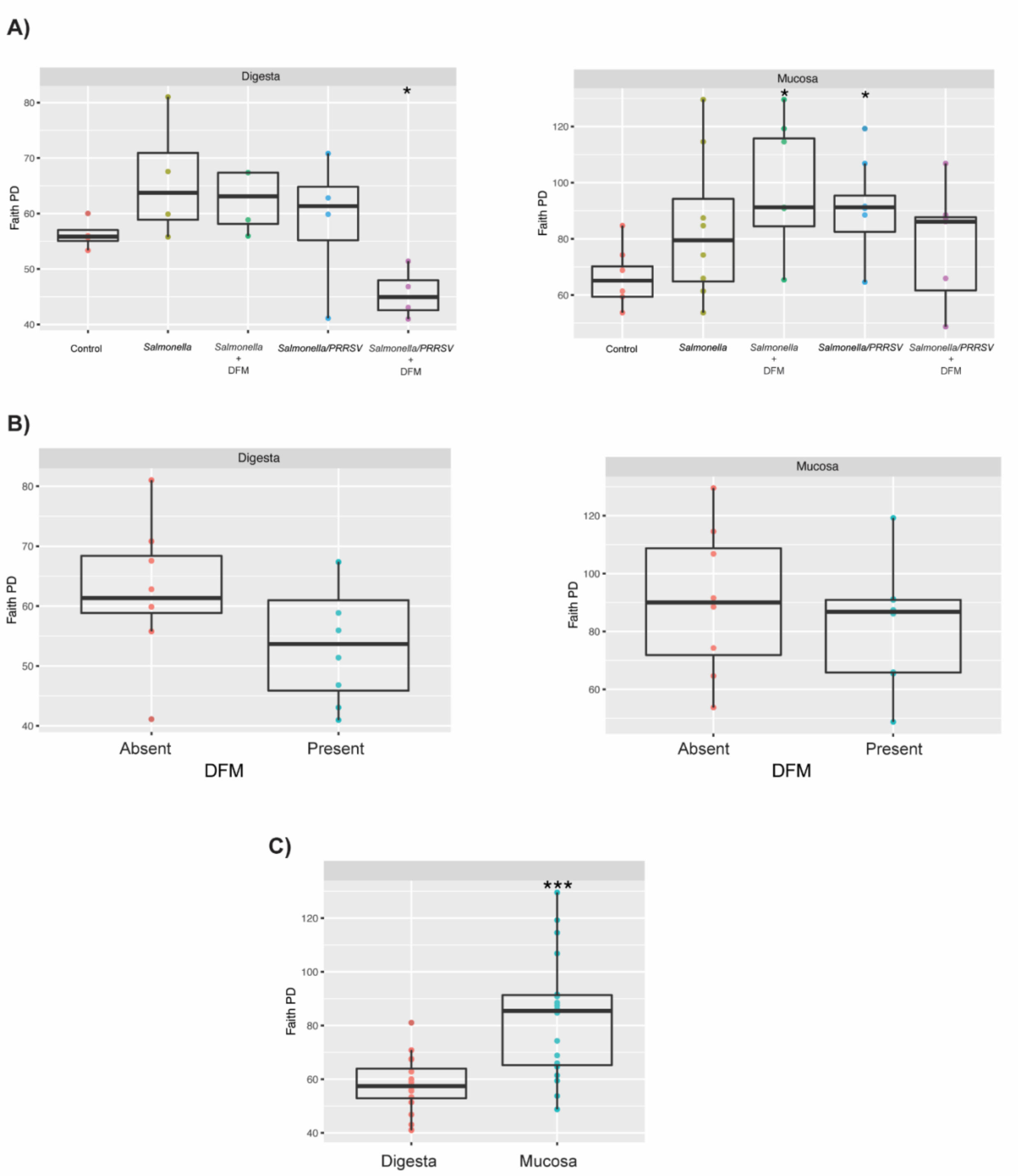

Figure S2. Box plots showing alpha diversity. Faith Phylogenetic Diversity (Faith PD) richness estimates (Y-axis) calculated from filtered and non-normalized feature data are plotted using phyloseq and ggplot packages. They are split (X-axis) by (A) treatment, (B) absence or presence of DFM and (C) sample types (digesta or mucosa) from all pigs. Lower and upper box edges correspond to the 25th and 75th percentiles. The black line between these quartiles represents the

median. The upper whisker extends from the box edge to the largest value no further than 1.5 * IQR from the box edge (where IQR is the inter-quartile range, or distance between the first and third quartiles). The lower whisker extends from the box edge to the smallest value at most 1.5 * IQR of the box edge. All data points beyond the whiskers are considered outliers. For significance calculation (*t*-test), control group, absence of DFM and digesta were set as reference in (A), (B) and (C), respectively. Significance is denoted by * ($p < 0.05$) and *** ($p < 0.001$).

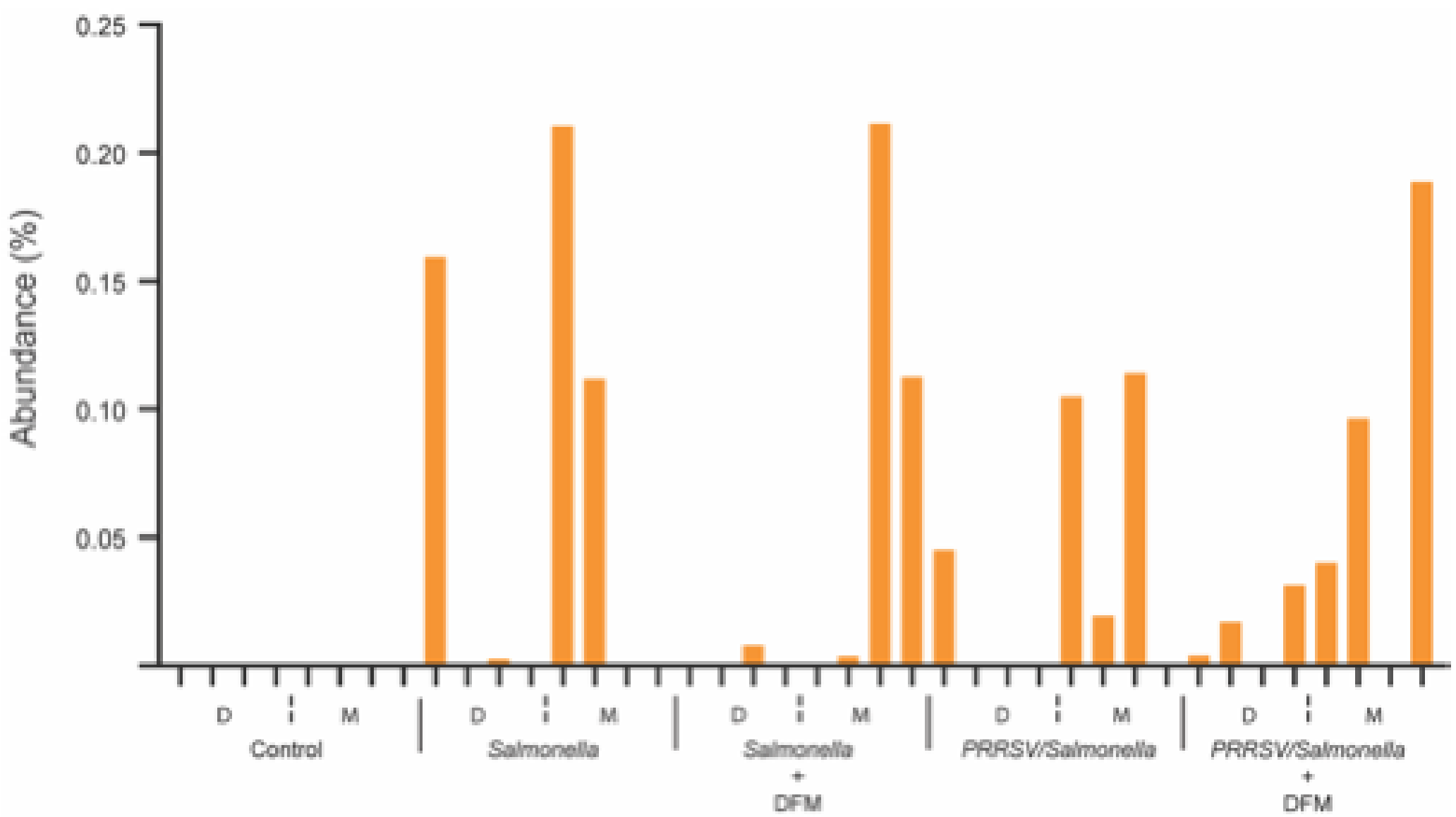

Figure S3. Relative abundance of *Salmonella enterica*. Relative abundance of *Salmonella enterica* based on 16S rRNA gene amplicon sequencing from digesta (D) and mucosa (M) samples from control pigs and the experimental pigs in the four treatment groups. Relative abundance of *Salmonella enterica* is presented as percentage of the total microbial population.

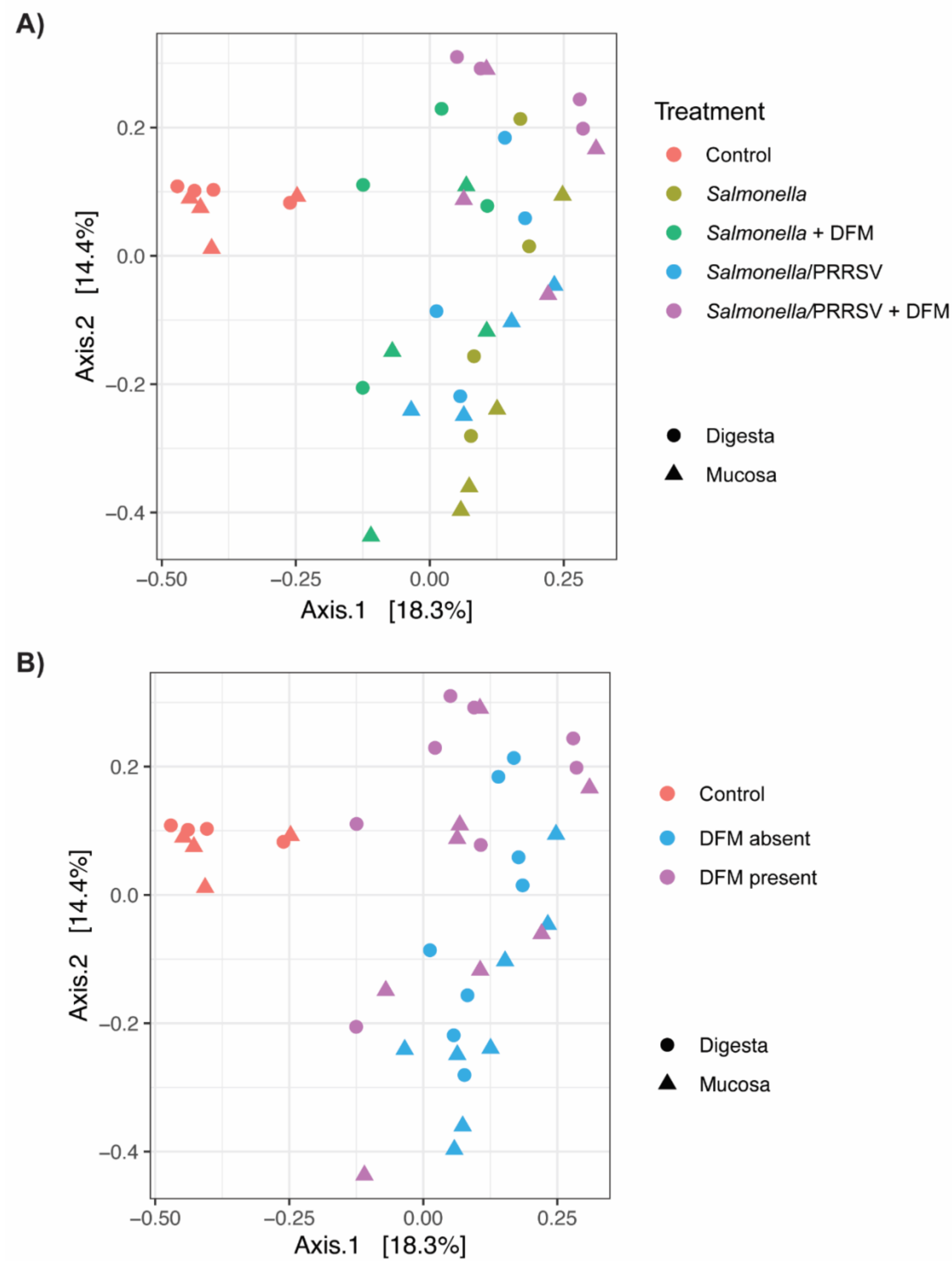

Figure S4. Principal coordinates analysis (PCoA). PCoA was performed on a Bray Curtis dissimilarity matrix for samples separated by (A) treatment and (B) presence and absence of DFM and plotted using the Phyloseq R package. Each shape represents a sample of either mucosa

(triangle) or digesta (circle) with color representing treatment type. Samples that are closer together are more similar than those that are ordinated further apart. Axes capture variation but are not correlated to variables.

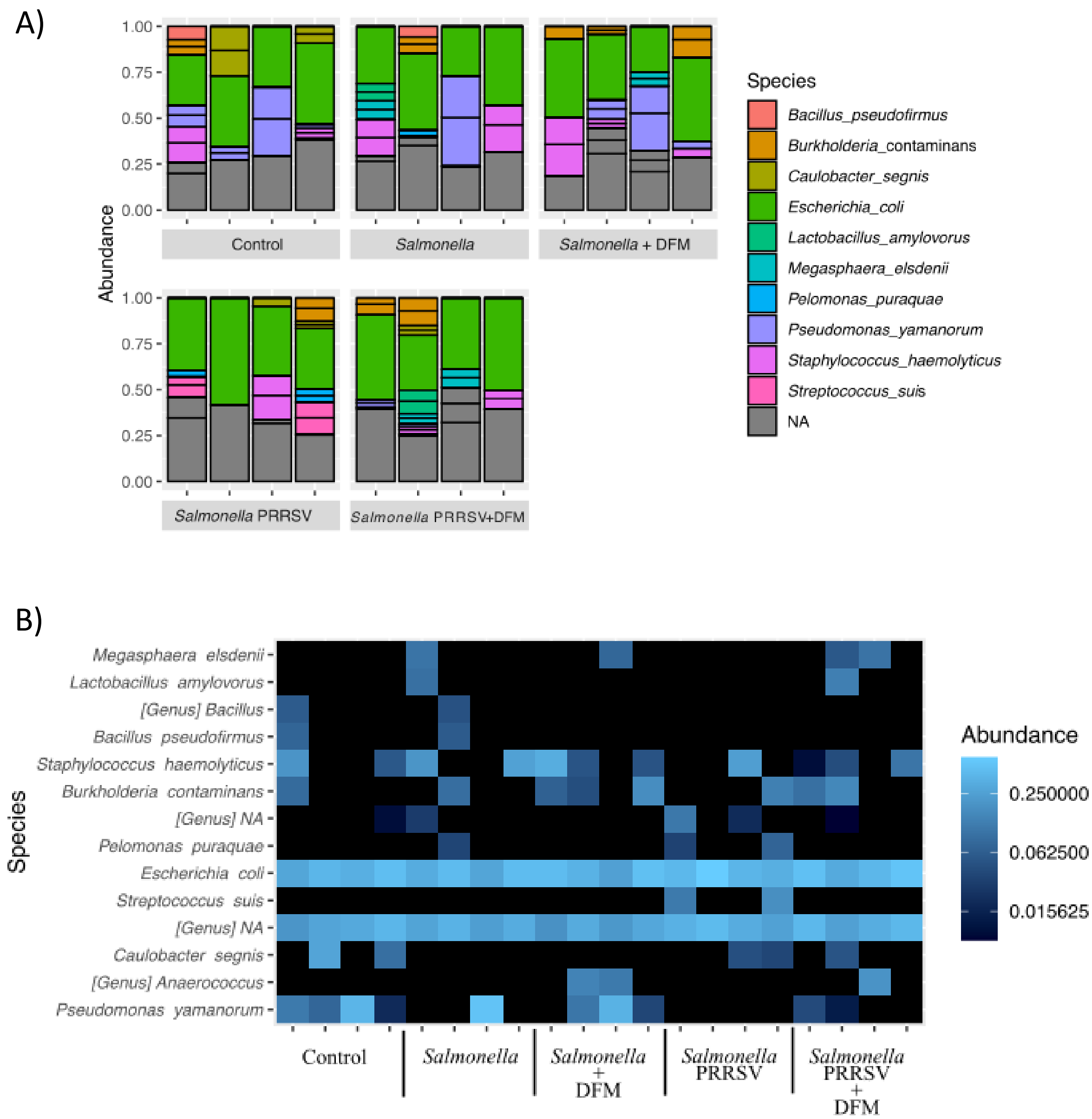

Figure S5. Relative abundance of bacterial species in lung tissue samples. Long-read amplicons were prepared with Shoreline Biome StrainIDä kit and were sequenced by PacBio Sequel II system. (A) Stacked bar charts showing the relative abundance of bacterial species in lung tissues. Bar height in each color represents the relative abundance of a bacterial species. NA refers to taxa not assigned at the genus level. (B) Heatmap representing the Bray-Curtis distances between samples, with bacterial species plotted on the Y-axis and treatment groups plotted on the X-axis. Lighter colors indicate more abundance of bacterial species. NA refers to taxa not assigned at the genus level.

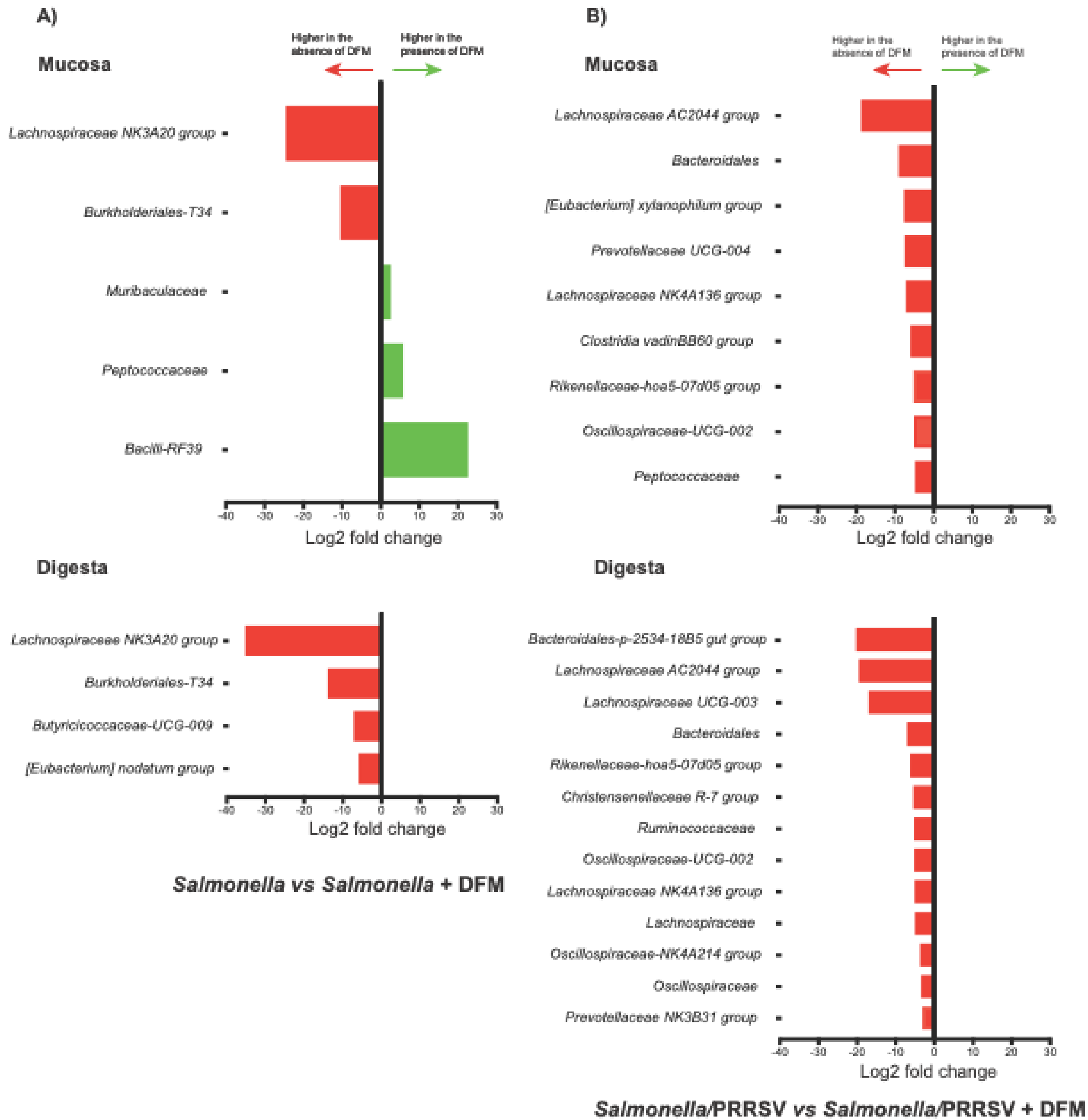

Figure S6. Differential abundance in the presence and absence of DFM. Log2 fold change for the abundance of bacteria (genus not classified) when comparing the microbial population in mucosa and digesta of (A) pigs challenged with *Salmonella* and fed with the DFM vs. pigs challenged with *Salmonella* in the absence of DFM and (B) pigs challenged with *Salmonella* plus PRRSV and fed with the DFM vs. pigs challenged with *Salmonella* plus PRRSV in the absence of DFM. Positive log2 fold change (green) indicates bacteria that are higher in the presence of DFM, whereas

negative log2 fold change (red) indicates bacteria that are higher in the absence of DFM. Only significant data with adjusted $p < 0.05$ are presented.

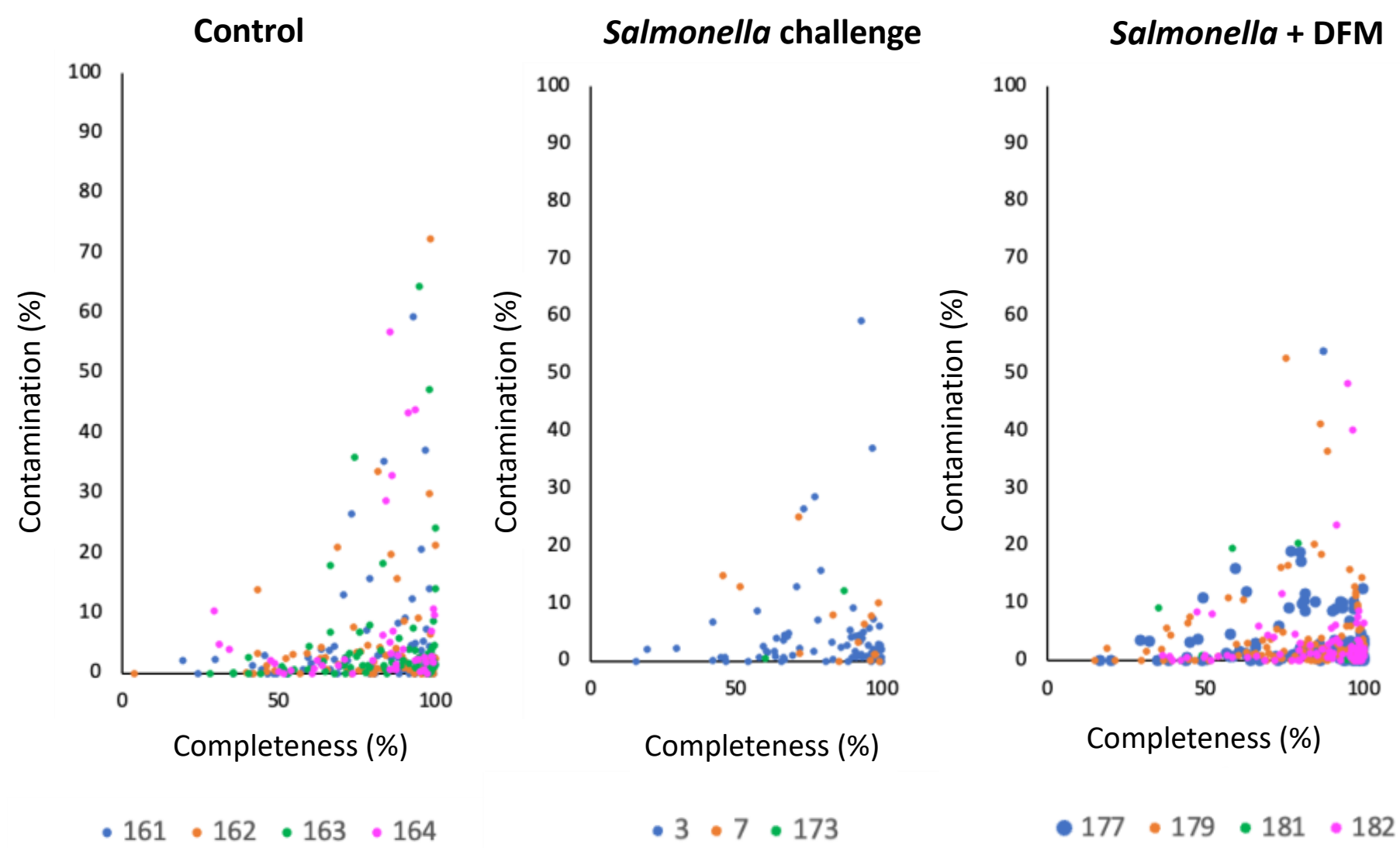

Figure S7. Distribution of the metagenome assembled genomes from the Control pigs (sample ID: 161, 162, 163, and 164), pigs challenged with *Salmonella* in the absence of DFM (3, 7, and 173), and pigs challenged with *Salmonella* and fed with the DFM (177, 179, 181, and 182) based on the percent of completeness and contamination.

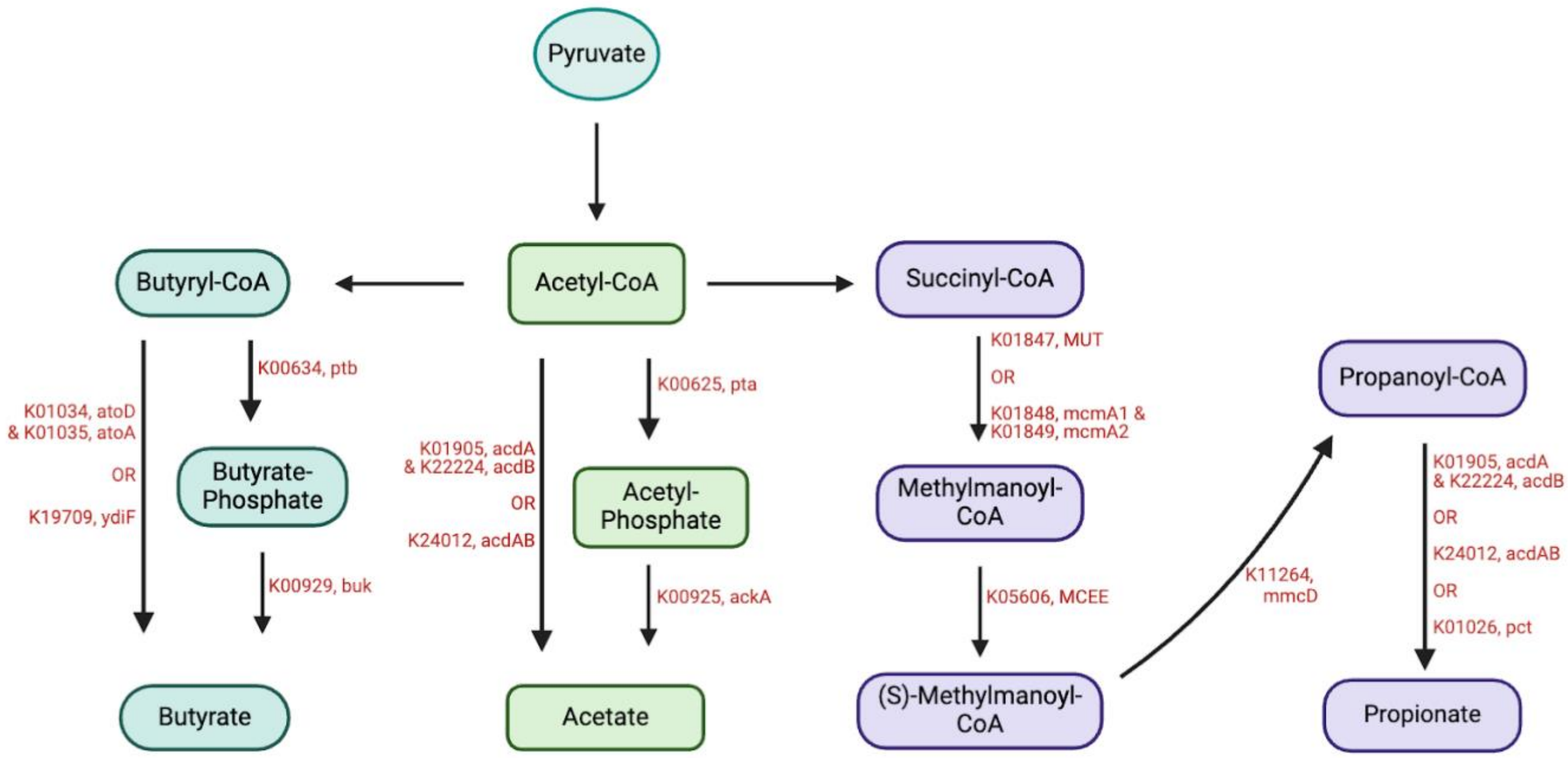

Figure S8. Pathways for butyrate, acetate, and propionate production are displayed with corresponding genes and associated Kegg numbers. Major intermediates are shown. Enzymes analyzed for butyrate production are butyryl-CoA:acetoacetate CoA transferase alpha subunit (atoD), butyryl-CoA:acetoacetate CoA transferase beta subunit (atoA), butyryl-CoA:acetate CoA-transferase (ydiF), phosphate butyryltransferase (ptb), and butyrate kinase (buk). Enzymes analyzed *only* for acetate production are phosphate acetyltransferase (pta) and acetate kinase (ackA). Enzymes analyzed *only* for propionate production are methylmalonyl-CoA mutase (MUT), methylmalonyl-CoA mutase, N-terminal domain (mcmA1), methylmalonyl-CoA mutase, C-terminal domain(mcmA1), methylmalonyl-CoA/ethylmalonyl-CoA epimerase (MCEE), methylmalonyl-CoA decarboxylase (mmcD and associated subunits), and propanoate CoA-transferase (pct). Enzymes analyzed for *both* acetate and propionate production include acetate:CoA ligase subunit alpha/propanoate:CoA ligase subunit alpha (acdA), acetate:CoA ligase subunit beta/propanoate:CoA ligase subunit beta (acdB), and acetate:CoA/propanoate:CoA ligase (acdAB).

Table S1. Experimental design of pigs challenged with either *Salmonella* or Porcine Reproductive and Respiratory Syndrome Virus (PRRSV) in the presence of a DFM cocktail

**Group #**	**Description**	**Mucosa Samples ID**				**Digesta samples ID**			
1	Control (No challenge – No DFM) group	113	114	115	116	161	162	163	164
2	*Salmonella* group	4	8	126	127	3	7	172	173
3	*Salmonella* + DFM group	131	133	135	136	177	179	181	182
4	PRRSV/*Salmonella* group	144	147	140	148	190	193	186	194
5	PRRSV/*Salmonella* + DFM group	154	157	152	160	198	201	196	204

Table S2. Digesta samples of the cecum in each treatment group used to obtain community genomic DNA

Group #	Description	Digesta samples ID			
1	Control (No challenge – No DFM)	161	162	163	164
2	*Salmonella* group	3	7	172	173
3	*Salmonella* + DFM group	177	179	181	182

Table S3. Total raw reads and trimmed sanitized reads of illumina sequencing in each treatment group

Treatment Groups	Sample_ID	Total Illumina PE Reads	Total Illumina Trimmed Sanitized Reads (no host)	Total ONP Reads	Total ONP Trimmed Sanitized Reads (no host)	% Host (Illumina)	% Host (ONT)	Comments
**1**	**161**	42,364,580	25,657,523	5,567,298	5,424,939	39.44%	2.56%	
**1**	**162**	36,371,081	20,610,395	6,448,615	6,171,748	43.33%	4.29%	
**1**	**163**	40,315,347	19,269,298	5,832,993	5,626,656	52.20%	3.54%	
**1**	**164**	40,317,956	20,171,450	5,410,326	5,014,470	49.97%	7.32%	
**2**	**3**	40,120,631	2,479,605	4,964,303	593,491	93.82%	88.04%	Significant amount of read loss during sanitation
**2**	**7**	39,174,843	1,656,806	5,358,152	191,627	95.77%	96.42%	Significant amount of read loss during sanitation
**2**	**172**	41,241,884	305,154	4,637,601	44,570	99.26%	99.04%	Significant amount of read loss during sanitation
**2**	**173**	42,186,648	2,040,205	9,018,352	411,959	95.16%	95.43%	Significant amount of read loss during sanitation
**3**	**177**	40,516,603	22,530,130	10,548,830	9,663,906	44.39%	8.39%	
**3**	**179**	40,623,913	18,489,151	3,854,728	3,801,863	54.49%	1.37%	
**3**	**181**	36,236,654	1,527,049	7,914,330	253,215	95.79%	96.80%	Significant amount of read loss during sanitation
**3**	**182**	45,758,974	17,627,205	4,866,113	4,449,269	61.48%	8.57%	

Table S4. Samples from the lung tissues collected for staining of thin sections and scoring for pathological findings

Pig ID	Treatment	Section	Sample Type	Blind ID
30	Control	Lung	Frozen lung section	E1
8	Control	Lung	Frozen lung section	E2
10	Control	Lung	Frozen lung section	E3
49	Control	Lung	Frozen lung section	E4
5	*Salmonella*	Lung	Frozen lung section	D1
3	*Salmonella*	Lung	Frozen lung section	D2
32	*Salmonella*	Lung	Frozen lung section	D3
4	*Salmonella*	Lung	Frozen lung section	D4
41	*Salmonella*/PRRS	Lung	Frozen lung section	C1
18	*Salmonella*/PRRS	Lung	Frozen lung section	C2
31	*Salmonella*/PRRS	Lung	Frozen lung section	C3
29	*Salmonella*/PRRS	Lung	Frozen lung section	C4
35	*Salmonella*/PRRS+DFM	Lung	Frozen lung section	B1
15	*Salmonella*/PRRS+DFM	Lung	Frozen lung section	B2
47	*Salmonella*/PRRS+DFM	Lung	Frozen lung section	B3
23	*Salmonella*/PRRS+DFM	Lung	Frozen lung section	B4
43	*Salmonella*+DFM	Lung	Frozen lung section	A1
33	*Salmonella*+DFM	Lung	Frozen lung section	A2
17	*Salmonella*+DFM	Lung	Frozen lung section	A3
9	*Salmonella*+DFM	Lung	Frozen lung section	A4

Table S5. Frequency of TIGRFAM domains and conserved sequence motifs among rSAM polypeptides identified in MAGs across all treatment groups. Only rSAM proteins were analyzed. TIGRFAM domains were annotated using the NCBI Conserved Domain Database, and conserved sequence motifs were extracted from ClustalW alignments of the rSAM sequences. The GGEPL motif associated with TIGR04068 was previously described by Hill et al. (2011) and Mantovani et al. (2015). Motifs with ≥75% conservation across aligned sequences are reported.

Domain or Motif Category	TIGRFAM Domain / Motif Description	Frequency (%)	Conserved Motif (≥75%)
**TIGR Domains**	TIGR04068 (Cys-rich peptide radical SAM maturase)	54.55	GGEPL
	TIGR03974 (SCIFF rSAM maturase)	51.82	Not described
	TIGR04085 (Radical SAM domain)	57.27	Not described
	TIGR04115 (Peptide maturation, sactipeptide-associated)	44.55	Not described
**rSAM Motifs**	rSAM N-terminal domain	92.73	CXXXCXXC
	SPASM domain (C-terminal extension)	56.36	CXXCXXXXXC
	Putative SAM-binding site	65.45	TNG
	Unknown function	47.27	SXDG

Table S6. Overview of treatment groups, including breed composition, sex, and body weight (PIC = Pig Improvement Company).

Group #	Treatment	Breed (PIC / Yorkshire)	Sex (Female / Male)	Starting Body Weight (kg)	Total Animals
1	Control	2 PIC / 2 Yorkshire	1 Female / 3 Males	5.61–9.45	4
2	*Salmonella*	3 PIC / 1 Yorkshire	0 Females / 4 Males	7.84–9.34	4
3	*Salmonella* + DFM	1 PIC / 3 Yorkshire	1 Female / 3 Males	5.53–7.61	4
4	*Salmonella*/PRRSV	2 PIC / 2 Yorkshire	2 Female / 2 Males	7.19–9.06	4
5	*Salmonella*/PRRSV + DFM	2 PIC / 2 Yorkshire	2 Female / 2 Males	5.8–9.1	4

Table S7. Ingredients and chemical composition of diet

Ingredient	Inclusion Weight (lbs) for 1 Ton of Complete Feed
Corn	840.40
Soybean Meal	630.00
Whey	400.00
Fat	60.00
Lime	24.00
Dical	14.00
Lysine	7.00
Swine Tm	7.00
Zinc Oxide	5.00
Salt-White	4.00
Vitmix ADEK	4.00
DL Methionine	3.00
Threonine	1.60

Table S8. Examples of sequences of known sactipepides

Type	Peptide name	Amino acid sequence
I	Subtilosin A	MKKAVIVENKGCATCSIGAACLVDGPIPDFEIAGATGLFGLWG
I	Thurincin H	METPVVQPRDWTCWSCLVCAACSVELLNLVTAATGASTAS
I	SKF	MKRNQKEWESVSKKGLMKPGGTSIVKAAGCMGCWASKSIAMTRVCALPHPA MRAI
I	Trn α	MEVMNNALITTKVDEEIGGNAACVIGCIGSCVISEGIGSLVGTAFTLG
I	Trn β	MEVLNKQNVNIIPESEEVGGWVACVGACGTVCLASGGVGTEFAAASYFL
I	Thuricin Z / Huazacin	MEPIQRDDYWGCALKCAGPCLGVCAIDTASPVMDAVGTASGYAGGHG
I	Hyicin 4244	MEQGVMVSNKGCSACAIGAACLADGPIPDEEVAGITGTFGIAS
II	RumC1	MRKIVAGKLQTGADFEGSKWGCVCSGSTAVANSHNAGPAYCVGYCGNNGVVT RNANANVAKTA
II	Streptosactin	MEKLQVIDLEELLEDSQGYVINASCGPSHSCGGGR
II	QmpA	MVVNTNKKYTEEGLLQTATNFCVNLCQMP
III	Enteropeptin	METALEEIKLIEKVEEMERKTEILEIVPTSVSEIVDSMKGRCPPSISGLVEIEQDL

Supplemental excel 1. Metadata for MAGs phylogenetic tree construction

Supplemental excel 2. Butyrate and propionate production related genes in microbial genomes in control, *Salmonella* challenge, and *Salmonella* challenge with DFM groups

Supplemental excel 3. Biosynthetic gene clusters in microbial genomes in control, *Salmonella* challenge, and *Salmonella* challenge with DFM groups

Supplemental excel 4. Bacteriocin related genes in microbial genomes in control, *Salmonella* challenge, and *Salmonella* challenge with DFM groups

Github file: containing commands used for analyses.

NextPolish:

```
$ nextPolish run.cfg
$ cat run.cfg
[General]
job_type = local
job_prefix = nextPolish
task = best
rewrite = yes
rerun = 3
parallel_jobs = 4
multithread_jobs = 3
genome = metaFlye_raw_assembly.fasta
genome_size = auto
workdir = ./01_rundir
polish_options = -p 1

[lgs_option] #optional
lgs_fofn = ./onp.fofn
lgs_options = -min_read_len 1k -max_depth 100
lgs_minimap2_options = -x map-ont
```

MetaBAT2:

```
$ jgi_summarize_bam_contig_depths --outputDepth genome.jgi.depth.txt
$SORTEDBAM
$ metabat2 -i $GENOMENAME -t $threads --unbinned --maxP 90 --minS 50 \
  -a genome.jgi.depth.txt -o metaBAT2_w_ShortReads/bin
```

MaxBin2:

```
$ /path/to/bin/run_MaxBin.pl -contig $CONTIGS -out $OUTPUTDIR/maxbin_out \
    -abund $ABUNDANCE -min_contig_length 1000 -markerset 40 -thread $THREADS
```

CheckM:

```
$ checkm lineage_wf -t $threads -x fa metaBAT2_w_ShortReads/
CheckM_w_ShortReads
$ checkm qa -t $threads -o 2 --tab_table -f CheckM_w_ShortReads/CheckM_qa_report.tsv
\
    CheckM_w_ShortReads/lineage.ms CheckM_w_ShortReads/
```

DASTool:

```
$ DAS_Tool -i $METABAT2,$MAXBIN2  -l metaBAT2,MaxBin2 -c $CONTIGS \
  -o $SAMPLEID/DASToolOut --threads $THREADS \
  --search_engine diamond --score_threshold 0.6
```

GTDBtk:

```
$ export GTDBTK_DATA_PATH=/path/to/data/GTDB/R207_v2/release207_v2

$ gtdbtk classify_wf --genome_dir $INPUTDIR --out_dir $OUTPUTDIR \
-x fa --prefix $SAMPLEID --cpus $SLURM_NPROCS \
--tmpdir $SCRATCH --scratch_dir $SCRATCH –debug
```

MetaGeneMark-2:

```
$ /path/to/bin/run_mgm.pl   --seq $GENOMENAME  \
    --out ${PREFIX}.MetaGeneMark2.annotation.gff3 --nt
${PREFIX}.MetaGeneMark2.nt \
    --aa  ${PREFIX}.MetaGeneMark2.aa  --format gff3

$ gffread -g polished_genome.fasta -y tmp_proteins.fa MetaGeneMark2.annotation.gff3
$ sed 's/\.$//g' tmp_proteins.fa | seqkit seq -g -w 0  > proteins.fasta
```

InterProScan:

```
$ interproscan.sh --tempdir ./tmp  -appl \

CDD,COILS,Gene3D,HAMAP,PANTHER,Pfam,PIRSF,PRINTS,PROSITEPATTERNS
,PROSITEPROFILES,SMART,SUPERFAMILY,TIGRFAM  \
        --cpu  $THREADS     -dp     -f  GFF3    -f  TSV  -goterms     -i  $proteins.fasta    -
o_output_iprscan
```
